# Genome-wide common and rare variant analysis provides novel insights into clozapine-associated neutropenia

**DOI:** 10.1038/mp.2016.97

**Published:** 2016-07-12

**Authors:** S E Legge, M L Hamshere, S Ripke, A F Pardinas, J I Goldstein, E Rees, A L Richards, G Leonenko, L F Jorskog, Clozapine-Induced Agranulocytosis Consortium, Jacqueline I Goldstein, L Fredrik Jarskog, Chris Hilliard, Ana Alfirevic, Laramie Duncan, Denis Fourches, Hailiang Huang, Monkol Lek, Benjamin M Neale, Stephan Ripke, Kevin Shianna, Jin P Szatkiewicz, Alexander Tropsha, Edwin JCG van den Oord, Ingolf Cascorbi, Michael Dettling, Ephraim Gazit, Donald C Goff, Arthur L Holden, Deanna L Kelly, Anil K Malhotra, Jimmi Nielsen, Munir Pirmohamed, Dan Rujescu, Thomas Werge, Deborah L Levy, Richard C Josiassen, James L Kennedy, Jeffrey A Lieberman, Mark J Daly, Patrick F Sullivan, K D Chambert, D A Collier, G Genovese, I Giegling, P Holmans, A Jonasdottir, G Kirov, S A McCarroll, J H MacCabe, K Mantripragada, J L Moran, B M Neale, H Stefansson, D Rujescu, M J Daly, P F Sullivan, M J Owen, M C O'Donovan, J T R Walters

**Affiliations:** 1 MRC Centre for Neuropsychiatric Genetics and Genomics, Division of Psychological Medicine and Clinical Neurosciences, School of Medicine, Cardiff University, Cardiff, UK; 2 Analytic and Translational Genetics Unit, Massachusetts General Hospital, Boston, MA, USA; 3 Stanley Center for Psychiatric Research, Broad Institute of MIT and Harvard, Cambridge, MA, USA; 4 Department of Psychiatry and Psychotherapy, Charité - Universitätsmedizin Berlin, Campus Mitte, Berlin, Germany; 5 Medical and Population Genetics Program, Broad Institute of MIT and Harvard, Cambridge, MA, USA; 6 Department of Psychiatry, University of North Carolina, Chapel Hill, NC, USA; 7 Psychiatric and Neurodevelopmental Genetics Unit, Massachusetts General Hospital, Boston, MA, USA; 8 King's College London, SGDP Centre, Institute of Psychiatry, Psychology and Neuroscience, De Crespigny Park, Denmark Hill, London, UK; 9 Discovery Neuroscience Research, Eli Lilly and Company Ltd, Lilly Research Laboratories, Erl Wood Manor, Surrey, UK; 10 Department of Psychiatry, University of Halle, Halle, Germany; 11 deCODE genetics, Sturlugata 8, Reykjavik, Iceland; 12 Department of Genetics, Harvard Medical School, Boston, MA, USA; 13 King's College London, Department of Psychosis Studies, Institute of Psychiatry Psychology and Neuroscience, London, USA; 14 Department of Psychiatry, University of Munich, Munich, Germany; 15 Department of Genetics, University of North Carolina, Chapel Hill, NC, USA; 16 Department of Medical Epidemiology and Biostatistics, Karolinska Institutet, Stockholm, Sweden

## Abstract

The antipsychotic clozapine is uniquely effective in the management of schizophrenia; however, its use is limited by its potential to induce agranulocytosis. The causes of this, and of its precursor neutropenia, are largely unknown, although genetic factors have an important role. We sought risk alleles for clozapine-associated neutropenia in a sample of 66 cases and 5583 clozapine-treated controls, through a genome-wide association study (GWAS), imputed human leukocyte antigen (HLA) alleles, exome array and copy-number variation (CNV) analyses. We then combined associated variants in a meta-analysis with data from the Clozapine-Induced Agranulocytosis Consortium (up to 163 cases and 7970 controls). In the largest combined sample to date, we identified a novel association with rs149104283 (odds ratio (OR)=4.32, *P*=1.79 × 10^−8^), intronic to transcripts of *SLCO1B3* and *SLCO1B7*, members of a family of hepatic transporter genes previously implicated in adverse drug reactions including simvastatin-induced myopathy and docetaxel-induced neutropenia. Exome array analysis identified gene-wide associations of uncommon non-synonymous variants within *UBAP2* and *STARD9*. We additionally provide independent replication of a previously identified variant in *HLA-DQB1* (OR=15.6, *P*=0.015, positive predictive value=35.1%). These results implicate biological pathways through which clozapine may act to cause this serious adverse effect.

## Introduction

Clozapine is the only licensed medication for treatment-resistant schizophrenia, defined as a failure to respond to at least two antipsychotic trials of sufficient dose and duration. Although it is the only treatment with proven efficacy in this severely impaired group of patients,^1, 2^ it is substantially under-prescribed^3^ due, at least in part, to the risk of haematological side effects of agranulocytosis and neutropenia (that is, reductions of neutrophils to levels below 500 or 1500 mm^−^^3^, respectively). The cumulative risk of agranulocytosis in those taking clozapine is 0.8% and for neutropenia is 2.9%.^4^ If undetected, compromised immune function secondary to agranulocytosis can be fatal, as happened in a series of patients when the drug was introduced in the 1970s, leading to its widespread withdrawal. Evidence of its marked effectiveness over other antipsychotics led to Federal Drug Agency (USA) approval in 1989 with stipulations about the need for regular blood monitoring to aid early detection of blood abnormalities. The requirement for blood monitoring limits the acceptability of the drug to patients, and poses an obstacle to its use in clinical practice.^5^

The aetiology of clozapine-induced blood disorders is currently unknown, although genetic causes contribute. The first genome-wide association study (GWAS) conducted by the Clozapine-Induced Agranulocytosis Consortium (CIAC) has provided substantial evidence for the role of *HLA-DQB1* and *HLA-B* in clozapine-associated neutropenia.^6^ In this study we report analyses incorporating GWAS, human leukocyte antigen (HLA) allele imputation, exome array and copy-number variation (CNV) to examine genetic associations with clozapine-associated neutropenia. Associated variants were combined in a joint meta-analysis with data from the CIAC study,^6^ giving the largest combined study sample of its kind to date.

## Materials and methods

### Sample description

Study individuals were from CLOZUK (*n*=5493) and CardiffCOGS (Cognition in Schizophrenia, *n*=156) samples. All had clinical or research diagnoses of schizophrenia.^7^ CLOZUK comprises individuals who were prescribed clozapine in the United Kingdom and have a clinical diagnosis of treatment-resistant schizophrenia.^7, 8^ The CLOZUK samples were acquired anonymously by the research team, in accordance with ethics permissions and the UK Human Tissue Act, in collaboration with Novartis, one of the UK suppliers of clozapine. Twelve months after sample acquisition, the research team was informed of those who had developed neutropenia while taking clozapine and, where available, the recorded lowest neutrophil counts of these individuals were supplied. CardiffCOGS is a schizophrenia sample recruited from secondary mental health services in South Wales, UK; for detailed sample description see.^8, 9^ As part of a comprehensive clinical interview, individuals were asked about lifetime clozapine use and occurrence of neutropenia. Clinical case notes were used to confirm neutropenia status, and lowest recorded neutrophil levels were collected.

Clozapine-associated neutropenia cases (*n*=66) developed an absolute neutrophil count (ANC) ⩽1500 mm^−^^3^ during treatment with clozapine. Following the approach of recent studies,^6, 10^ we assessed cases with neutropenia because the success of the monitoring system and preemptive drug withdrawal in the United Kingdom has made agranulocytosis extremely rare. This neutrophil count threshold is used in the United Kingdom as a trigger to discontinue clozapine. Controls (*n*=5583) had received clozapine for a minimum of a year without developing an ANC <2000 mm^−^^3^. Those who had a test result (1500 mm^−^^3^<ANC<2000 mm^−^^3^) were excluded from all analyses (*n*=20). No differences in age or sex were observed between clozapine-associated neutropenia cases and controls (Supplementary Table 1). All individuals were of European ancestry, as determined by self-report and principal component analysis (PCA) of GWAS data.

### Genotyping

Genotyping was performed at the Broad Institute, Cambridge, MA, USA. CardiffCOGS and part of the CLOZUK sample (40 cases and 3573 controls) were genotyped on Illumina HumanOmniExpressExome-8v1 and the remainder of the CLOZUK sample (26 cases and 2098 controls) were genotyped on both Illumina HumanOmniExpress-12v1 and Illumina HumanExome BeadChip (San Diego, CA, USA).

### Genome-wide association study

Quality-control procedures and imputation were conducted using the Psychiatric Genomics Consortium pipeline.^7^ Imputation was performed using IMPUTE2 (ref. 11) and a reference panel from the full 1000 Genomes Project data set (freeze date August 2012, see Supplementary Methods). Principal component estimation was conducted using EIGENSTRAT to exclude outliers and assess population stratification^12^ (Supplementary Figures 1 and 2). We included genotyping array as well as the first three principal components as covariates to account for population structure. Single-nucleotide polymorphisms (SNPs) with allele frequencies that differed between genotyping arrays at *P*<1 × 10^−5^ were excluded (Supplementary Figure 3). We selected common SNPs for analysis with high imputation quality (imputation INFO score≥0.8, minor allele frequency (MAF) ≥0.01 in cases and controls). Association analysis was performed using logistic regression in PLINK^13^ and SNPs functionally annotated using the Scripps genome advisor.^14^ PLINK^13^ was used to identify index SNPs in relative linkage equilibrium. Further details of quality-control procedures and statistical analyses are provided in Supplementary Methods.

### *HLA* analysis

Classical *HLA* alleles and amino-acid polymorphisms were imputed using SNP2HLA (version 1.02)^15^ using BEAGLE (version 3.0.4)^16^ from genotyped common variants using a reference data set of 5225 individuals from Type 1 Diabetes Genetics Consortium (T1DGC). We used the same procedures for SNP selection, analysis and covariate selection (three PCAs derived from GWAS, plus genotyping array) as described for the GWAS analysis above. Owing to complex and extended linkage disequilibrium (LD) in the major histocompatibility complex, we did not identify index SNPs in relative linkage equilibrium.

We additionally genotyped a candidate SNP, *HLA-DQB1* 6672G>C (ref. 17; rs113332494) in 60 cases and 305 age- and sex-matched controls. This SNP was genotyped separately as it was not imputed with sufficient quality to be reported in the GWAS or HLA analyses, and was a strong candidate variant.^17^ Genotyping was conducted at deCODE genetics using the Centaurus (Nanogen) platform.^18^ Association with clozapine-associated neutropenia was tested using Fisher’s exact test, given low minor allele counts; however, to ensure there was no effect of population stratification, we also conducted a logistic regression including three PCAs derived from GWAS with 5 × 10^8^ permutations to generate empirical *P*-values.

### Exome array analysis

The Illumina exome array is designed to genotype uncommon-to-rare coding variants previously observed in whole-exome sequencing studies. Exome array data were available for 57 cases and 4958 exposed controls. Full details of the quality-control procedures are provided in an open access publication^19^ and Supplementary Methods. PCA was conducted using EIGENSTRAT,^12^ with 14 743 common exome array variants in relative linkage equilibrium (MAF⩾0.05, *r*^2^<0.2) to assess population structure and identify outliers (Supplementary Figure 4). Owing to the relatively small case sample size in our study we did not apply a frequency filter to variants in this analysis. Single variant association was conducted using logistic regression in PLINK with the first 10 principal components included as covariates. Adaptive permutations (between 10 and 1 × 10^9^) were used to generate empirical *P*-values in logistic regression analyses. PLINK^13^ was used to identify index SNPs in relative linkage equilibrium. To test for the effects of multiple functional variants in genes, we used SKAT-O (ref. 20) with 2 × 10^6^ permutations, including the first 10 principal components, for genes with at least two uncommon (MAF<0.05), non-synonymous (missense, stop or splice) variants.

### Copy-number variation

The identification and quality control of CNV for this sample has been previously described^8^ and is detailed in Supplementary Methods. CNVs were included if they had a frequency ⩽0.01, contained ⩾10 probes and were ⩾100 kb in length. Samples that passed both CNV and GWAS quality control (63 cases and 5456 controls) were used to test genes for enrichment of exon disrupting CNVs using a two-sided Fisher’s exact test. Deletions and duplications were analysed separately.

### Secondary analysis of clozapine-associated neutropenia below ⩽1000 mm^−^^3^

We conducted secondary analyses on a subset of the more severely affected cases with ANC ⩽1000 mm^−^^3^ (*n*=18). A total of four of these cases had developed agranulocytosis (ANC ⩽500 mm^−3^). We assessed the association of single variants with clozapine-associated neutropenia below ⩽1000 mm^−^^3^ in GWAS (*N*=18), exome array (*N*=16) and HLA imputation (*N*=18) analyses. All analyses conducted were consistent with methods used for clozapine-associated neutropenia, described above.

### Replication sample and meta-analysis

We obtained summary statistics for associated SNPs from a recently published study by the CIAC.^6^ In this study, Goldstein *et al.*^6^ conducted a comprehensive genetic association study in 163 clozapine-induced neutropenia cases (98 with ANC <500 mm^−3^, 61 with 500⩽ANC⩽1000 mm^−3^ and 4 with ANC ⩽1500 mm^−3^). The CIAC study included the following: GWAS (161 cases with clozapine-induced neutropenia, 249 clozapine-exposed controls without neutropenia and 947 unexposed controls), exome array analysis (148 cases and up to 7970 unexposed controls) and classical *HLA* allele imputation (162 cases and 4319 unexposed controls). These data sets formed the replication samples for the current study and were combined with results for both (i) clozapine-associated neutropenia and (ii) neutropenia ⩽1000 analyses. SNPs that were associated with clozapine-associated neutropenia at *P*<1 × 10^−4^ from our GWAS, or *P*<0.05 from our *HLA* variant analysis, were combined with the replication data in fixed-effects meta-analyses using PLINK to estimate a combined odds ratio (OR) weighted by the study’s inverse standard error (s.e.). If an index SNP was not present in the replication data, a proxy SNP in strong LD (*r*^2^⩾0.8) was substituted and the s.e. weighted (s.e.w.) to account for the lack of information: s.e.w.=s.e./sqrt(*r*^2^).^21^ The variants that were associated with clozapine-associated neutropenia from exome array analyses with *P*<0.01 were combined with the replication data in a *P*-value-based method in METAL,^22^ weighted by the square root of the total sample size. We used different *P*-value replication thresholds for the *HLA* and exome array to arrive at approximately the same number of variants.

## Results

**Figure 1:**
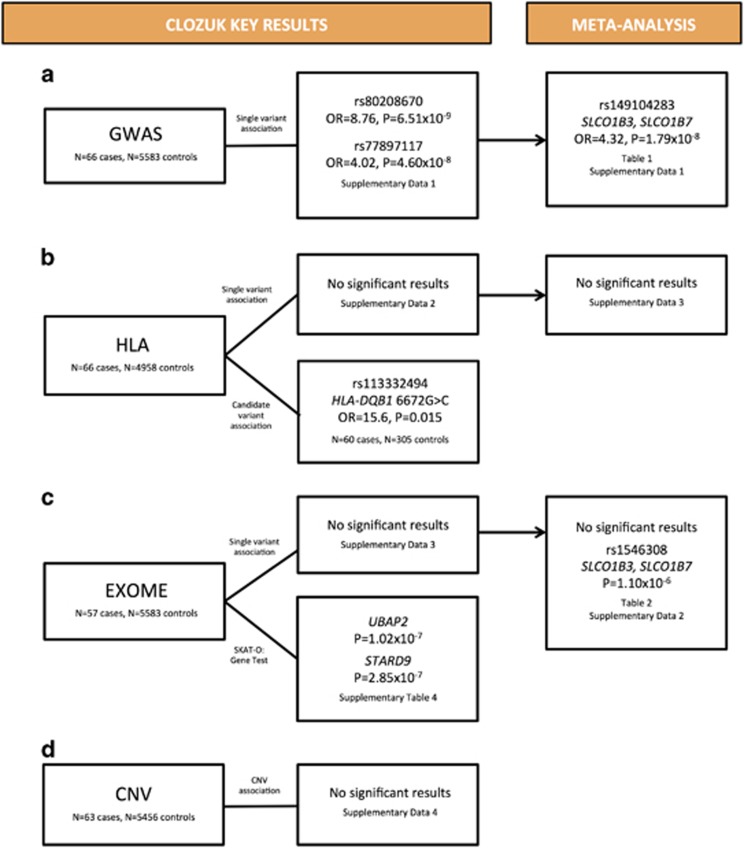
Study design and key results. To investigate the association of genetic variants with clozapine-associated neutropenia, we conducted (**a**) a genome-wide association study (GWAS), (**b**) human leukocyte antigen (HLA) allele imputation and genotyped a candidate variant of interest, HLA-DQB1 6672G>C/ rs113332494, (**c**) exome array single variant and gene-based analysis and (**d**) copy-number variation (CNV) analysis. We then took forward the associated variants from GWAS, HLA and exome array analyses to a combined meta-analysis with the Clozapine-Induced Agranulocytosis Consortium (CIAC) study.

Figure 1 provides a summary of the study design and key results from each analysis.

### Genome-wide association study

We performed a GWAS of 7 559 010 genotyped and imputed common SNPs (QQ plot in Supplementary Figure 5, *λ*_GC_=0.95). Two SNPs were associated with clozapine-associated neutropenia at the genome-wide significant level of *P*<5 × 10^−8^ (Supplementary Figure 6 and Supplementary Data 1); rs80208670 on chromosome 13 (OR=8.76, 95% confidence interval (CI): 4.21–18.25, *P*=6.51 × 10^−9^) and rs77897117 on chromosome 1 (OR=4.02, 95% CI: 2.46–6.57, *P*=4.60 × 10^−8^). Our sample size had 80% power to detect an OR>4 for alleles with MAF>0.10 at *P*<5 × 10^−8^ (Supplementary Figure 7). The genome-wide significant SNPs from the discovery CLOZUK GWAS, rs80208670 and rs77897117, were not significantly associated in CIAC (OR=1.69, *P*=0.27 and OR=0.67, *P*=0.28, respectively).

In total, there were 266 independent (*r*^2^<0.1) SNPs associated with clozapine-associated neutropenia at *P*<1 × 10^−4^ and we sought replication of these SNPs in the CIAC sample (257 of these SNPs were available or had an appropriate proxy, Supplementary Data 1). Table 1 lists the 10 most strongly associated SNPs from the meta-analysis. One SNP on chromosome 12 surpassed the GWS threshold for association with clozapine-associated neutropenia (OR=4.32, *P*=1.79 × 10^−8^). rs149104283 is intronic to transcripts of *SLCO1B3* and *SLCO1B7* (solute carrier organic anion transporter family, member 1B3 and member 1B7) and was present in 7.37% of cases versus 1.52% of controls in our sample and 4.20% of cases versus 1.67% of controls in the CIAC sample. For consideration of how this may be translated to risk allele carrier status see Supplementary Results.

### *HLA* analysis

No imputed classical *HLA* allele or amino-acid polymorphism was associated with clozapine-associated neutropenia at the genome-wide significant level (*P*<5 × 10^−8^) in either the discovery analysis (SNPs=7751) or combined meta-analysis (SNPs=102; Supplementary Data 2). It was not possible to impute the amino-acid polymorphisms *HLA-DQB1* (126Q) and *HLA-B* (158T) implicated in the CIAC study^6^ with sufficient quality (INFO>0.8) in the discovery sample.

We additionally genotyped a previously associated variant, *HLA-DQB1* 6672G>C ^17^ (rs113332494), in 60 cases and 305 age- and sex-matched controls as it was not imputed with sufficient quality. We found independent support for association of *HLA-DQB1* 6672G>C (OR=15.6, 95% CI: 1.6–151.4, *P*=0.015), replicating previous reports of association with clozapine-induced agranulocytosis.^17^ The association strengthened when considering only those with ANC below ⩽1000 mm^−^^3^ (OR=38.1, 95% CI: 3.4–430.9, *P*=0.0079). For the associated ‘G’ allele, we found three heterozygote carriers among 60 cases, and a single heterozygote in 305 controls. Lowest neutrophil counts were available for two of the three ‘G’ case carriers, both of whom had a neutrophil level <1000 mm^−^^3^ (700 and 900). The association remained after adjusting for GWAS PCAs. For further consideration of possible population-specific effects at this locus please refer to Supplementary Results.

### Exome array analysis

In exome array analyses, no single variant exceeded a significance threshold of *P*<4.3 × 10^−7^, corresponding to a Bonferroni correction for 115 000 variants tested, in either the discovery or combined meta-analyses (Supplementary Data 3, QQ plot given in Supplementary Figure 8, *λ*_GC_=1.11). Table 2 lists the 10 most strongly associated exome variants from the meta-analysis. Of interest is rs1546308 (*P*=1.10 × 10^−6^), a missense variant in *SLCO1B7* and intronic to *SLCO1B3,* that is 92 kb from the SNP that emerged as the only genome-wide significant variant from GWAS meta-analysis. rs1546308 is predicted to be benign and was present in 15.8% of cases versus 5.8% of controls in our sample and 9.3% of cases versus 5.6% of controls in the CIAC sample.

Supplementary Table 3 displays the 10 most significantly associated genes (including the total allele count and number of variants contributing to each gene) based on the SKAT-O analysis of genes with at least two uncommon (MAF<0.05), non-synonymous variants from the exome array. There was evidence of association for two genes that exceeded a threshold of *P*<2.5 × 10^−6^ (corresponding to a Bonferroni correction of 20 000 genes tested;^23^ QQ plot in Supplementary Figure 9): *UBAP2* on chromosome 9 (*P*=1.02 × 10^−7^) and *STARD9* on chromosome 15 (*P*=2.85 × 10^−7^). Owing to differing analytical methods used by CIAC, it was not possible to combine our gene-based results in a joint analysis (see Supplementary Methods for detailed explanation).

### Copy-number variation

In a genome-wide analysis, no individual gene was significantly enriched for large, rare exonic CNVs that exceeded a significance threshold of *P*<2.5 × 10^−6^ (Supplementary Data 4).

### Associations with rs1546308 and rs149104283 are not independent

**Figure 2:**
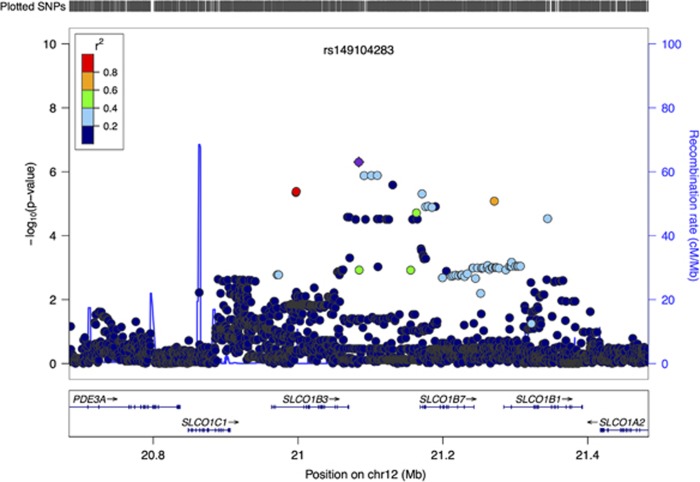
Association of 12p12.2 with clozapine-associated neutropenia. LocusZoom plot of the region associated with clozapine-associated neutropenia on chromosome 12p12.2 in CLOZUK (discovery) sample. Genes within the region are shown in the lower panel, and the unbroken blue line indicated the recombination rate within the region. Each circle represents the *P*-value for one SNP in the discovery sample, with the top SNP rs149104283 shown in purple and the SNPs in the region coloured depending on their degree of correlation (*r*^2^) with rs149104283 (as estimated by LocusZoom on the basis of CEU HapMap haplotypes).

We investigated the independence of rs1546308 (missense variant in *SLCO1B7* and intronic in *SLCO1B3* from exome chip analysis) and rs149104283 (GWAS intronic variant in *SLCO1B3* and *SLCO1B7*) in samples with data available for both variants (55 cases and 4834 controls). The LD between the two variants in the sample was *r*^2^=0.15, *D*’=0.84. In a conditional logistic regression, the strength of the association of rs1546308 with clozapine-associated neutropenia was attenuated from OR=3.00 (95% CI: 1.735–5.189, *P*=8.40 × 10^−5^) to OR=2.16 (95% CI: 1.093–4.251, *P*=0.027) after adjusting for rs149104283. Haplotype analysis did not strengthen the association signal. Thus, these two findings are not independent, and the associated region spans *SLCO1B1*, *SLCO1B3* and *SLCO1B7* (Figure 2 and Supplementary Figure 14).

### Secondary analysis of clozapine-associated neutropenia below ⩽1000 mm^−^^3^

Results of the GWAS for the more stringent neutropenia cutoff of ⩽1000 mm^−^^3^ are presented in Supplementary Results. The combined GWAS meta-analysis identified no loci exceeding genome-wide significance. The genome-wide significant SNP from the GWAS meta-analysis, rs149104283, had a MAF of 7.8% in cases with ANC ⩽1000 mm^−3^, and was associated at OR=7.6, *P*=0.0027 in the discovery sample. No variants exceeded relevant significance thresholds in imputed HLA and exome array discovery or combined meta-analyses (see Supplementary Results).

## Discussion

We have conducted a multifaceted genetic analysis of clozapine-associated neutropenia in the largest combined sample studied to date. Using GWAS, we identify a novel association implicating a family of organic anion transporters involved in drug metabolism, which have been previously associated with adverse drug reactions. We also found evidence for effects of uncommon non-synonymous variants within *UBAP2* and *STARD9* and provide independent replication of a previously identified variant in *HLA-DQB1*.

The primary GWAS finding from the meta-analysis was a genome-wide significant association with neutropenia for rs149104283. The association effect size of this polymorphism is larger in the CLOZUK discovery sample (OR=6.2) than in the CIAC replication data set (OR=2.95), as would be expected from winner’s curse. It follows that the true effect size probably lies closer to the CIAC estimate, although this requires confirmation using independent data. rs149104283 is an intronic SNP for transcripts of both *SLCO1B3* and *SLCO1B7*—the associated region containing a third member of this organic anion transporter family*, SLCO1B1. SLCO1B7* encodes a putative protein (OAT1B7) that is poorly characterised, based on coding sequence prediction, and its functionality is unknown. *SLCO1B3* and *SLCO1B1* share sequence homology and encode liver-specific organic anion transporter polypeptides (OATP1B3 and OATP1B1) that are multipass transmembrane proteins expressed exclusively in the basolateral membrane of hepatocytes.^24^ They facilitate uptake of exogenous substances, including drugs, from the portal vein into hepatocytes, where the substance is subsequently modified either via metabolism with cytochrome (CYP) 450 enzymes or excreted.^25^

Polymorphisms in *SLCO1B1* and *SLCO1B3* have been implicated in adverse reactions with other drugs. In 2008, a GWAS identified a missense variant rs4149056 in *SLCO1B1* that increased the risk of simvastatin-induced myopathy by increasing the area under the curve for simvastatin, particularly in those taking high doses.^26^ This prominent pharmacogenetic finding has been widely replicated and has led to recommendations for its use as a routine preemptive clinical test.^27^ Particularly relevant to the current study are reports of an association between rs11045585, an intronic variant in *SLCO1B3,* and severe leukopenia/neutropenia induced by docetaxel, a chemotherapeutic agent,^28^ and that this may be secondary to alterations in the pharmacokinetics and bioavailability of the drug.^29, 30, 31^ These polymorphisms were not in high LD (*r*^2^<0.1 for both) with the index SNP in this study, although rs11045585 was weakly associated with neutropenia in our discovery sample (OR=1.62, *P*=0.03).

Together, the findings suggest the hypothesis that genetic variants at *SLCO1B3* (and/or *SLCO1B1*) increase the risk of clozapine-associated neutropenia through a pharmacokinetic mechanism. It is unclear whether clozapine plasma levels are associated with the development of neutropenia.^32, 33, 34^ One of the best-supported hypotheses to explain clozapine’s association with agranulocytosis relates to the bioactivation of clozapine, or a stable metabolite, to a chemically reactive nitrenium ion.^35^ The propensity for nitrenium ions to cause apoptosis to neutrophils, or be toxic to neutrophil precursors, is dose-dependent, lending support to the hypothesis that clozapine pharmacokinetics and bioavailability are related to its potential to cause neutropenia.^36, 37^

In analysis of exome chip data we found evidence of association with neutropenia for uncommon non-synonymous variants in *STARD9* and *UBAP2*. *STARD9* is a mitotic kinesin, and *STARD9*-depleted cancer cells have abnormal cellular morphology and undergo apoptosis.^38^ In addition, STARD9 depletion was found to synergise with the chemotherapeutic agent taxol, the use of which is dose-limited because of neutropenia.^38^ The function of *UBAP2* is undetermined, although it has an ubiquitin-associated domain and is widely expressed across tissues including bone marrow. The ubiquitination pathway has been shown to modulate the granulocyte colony-stimulating factor receptor,^39, 40^ a critical regulator of neutrophil production. A recent study reported the association of a missense variant in the ubiquitin gene *USP43* with clozapine-associated neutropenia.^10^

Our final finding adds to the growing evidence implicating *HLA-DQB1* in clozapine-associated neutropenia, supporting the recently published CIAC study.^6^ There have been further reports implicating SNPs within *HLA-DQB1*,^17, 41, 42^ although these samples and those in CIAC are overlapping; thus, we believe we provide the first fully independent replication implicating this locus in clozapine-associated neutropenia/agranulocytosis. The *HLA-DQB1* variant alone has a positive predictive value of 35.1% (see Supplementary Table 4). Although this is promising, the majority of those that develop neutropenia or agranulocytosis while taking clozapine are not carriers of this risk allele, or indeed the other alleles we have identified in this study. The sensitivity for a test including rs149104283 (GWS intronic variant in *SLCO1B3* and *SLCO1B7*), rs1546308 (missense variant in *SLCO1B7*) and rs113332494 (*HLA-DQB1*) is 29.17%, the specificity 90.61%, the positive predictive value 9.94% and the negative predictive value 97.30% (Supplementary Table 4). Although the variants identified in this study convey a substantially increased risk for clozapine-associated neutropenia, they are currently on their own unlikely to have clinical utility for pharmacogenetic testing,^43^ particularly as there is currently no alternative treatment for those with treatment-resistant schizophrenia.

An important consideration is that the majority of cases in our analyses had developed neutropenia rather than agranulocytosis. It is now very rare in the United Kingdom to develop agranulocytosis because of the success of the monitoring system and the fact that clozapine is stopped once neutropenia is detected; in fact, only four cases met this threshold in our sample. Despite this, we found that the major findings from our neutropenia analysis extended to the secondary analyses, which was restricted to those with an ANC⩽1000 mm^−3^, indicating that the clozapine-associated neutropenia findings are likely to be applicable to those with severe neutropenia and agranulocytosis.

Our findings provide novel insights into putative biological processes underlying clozapine-associated neutropenia. Furthermore, we have indicated a potential link between the pharmacokinetics of clozapine and risk of neutropenia/agranulocytosis with potentially important clinical implications. The development of such understanding should help widen the availability of clozapine with a beneficial impact on those with treatment-resistant schizophrenia.

## Clozapine-induced Agranulocytosis Consortium (CIAC) members

Jacqueline I Goldstein, SB; L Fredrik Jarskog, MD; Chris Hilliard, BS; Ana Alfirevic, PhD; Laramie Duncan, PhD; Denis Fourches, PhD; Hailiang Huang, PhD; Monkol Lek, PhD; Benjamin M Neale, PhD; Stephan Ripke, MD; Kevin Shianna, PhD; Jin P Szatkiewicz, PhD; Alexander Tropsha, PhD; Edwin JCG van den Oord, PhD; Ingolf Cascorbi, MD, PhD; Michael Dettling, MD, PhD; Ephraim Gazit, MD, PhD; Donald C Goff, MD; Arthur L Holden, MBA; Deanna L Kelly, PharmD, BCPP; Anil K Malhotra, MD; Jimmi Nielsen, PhD; Munir Pirmohamed, MD, PhD; Dan Rujescu, MD, PhD; Thomas Werge, MSc, PhD; Deborah L Levy, PhD; Richard C Josiassen, PhD; James L Kennedy, PhD; Jeffrey A Lieberman, MD; Mark J Daly, PhD; Patrick F Sullivan, MD, FRANZCP.

## Figures and Tables

**Table 1:** GWAS meta-analysis top 10 SNPs

*CHR*	*SNP*	*Position*	*A1*	*CLOZUK*			*CIAC*			*Meta-analysis*		*Nearest gene*	*Location*
				P*-value*	*OR*	*INFO*	P*-value*	*OR*	*INFO*	P*-value*	*OR*		
12	rs149104283	21 083 862	T	4.98 × 10^−7^	6.20	0.87	3.61 × 10^−3^	2.95	0.87	1.79 × 10^−8^	4.32	*SLCO1B3, SLCO1B7*	Intronic, intronic
13	rs80208670	84 438 088	C	6.51 × 10^−9^	8.76	0.83	0.2728	1.69	0.83	1.56 × 10^−7^	4.69	*SLITRK1*	13 kb Downstream
1	rs184597564	82 236 406	A	2.48 × 10^−5^	4.03	0.89	4.99 × 10^−3^	2.40	0.94	8.01 × 10^−7^	3.06	*ADGRL2*	Intronic
7	rs78900159	76 968 378	A	6.94 × 10^−6^	4.01	0.99	0.0247	1.97	0.98	2.02 × 10^−6^	2.79	*GSAP*	Intronic
10	rs16916041	63 146 547	T	7.40 × 10^−6^	2.57	1.02	0.0205	1.58	0.96	2.05 × 10^−6^	1.98	*TMEM26*	20 kb Downstream
16	rs11649311	25 226 020	T	9.70 × 10^−5^	2.07	0.89	2.62 × 10^−3^	1.51	0.89	2.26 × 10^−6^	1.68	*AQP8*	2 kb Upstream
17	rs117202297	53 769 035	T	8.48 × 10^−6^	6.25	0.82	0.1503	2.66	0.66	5.27 × 10^−6^	4.97	*TMEM100*	28 kb Downstream
17	rs80282661	13 252 073	T	1.99 × 10^−6^	6.15	0.90	0.2632	1.85	0.89	5.49 × 10^−6^	4.17	*HS3ST3A1*	147 kb Downstream
1	rs185053659	60 704 250	A	7.76 × 10^−6^	5.80	0.86	0.1012	2.13	0.92	8.05 × 10^−6^	3.80	*C1orf87*	165 kb Upstream
14	rs78074145	40 404 458	C	1.13 × 10^−6^	4.05	0.96	0.2716	1.44	0.99	1.08 × 10^−5^	2.60	*FXB033*	503 kb Upstream

Abbreviations: A1, minor reference allele; CHR, chromosome; CIAC, Clozapine-Induced Agranulocytosis Consortium; GWAS, genome-wide association study; OR, odds ratio; SNP, single-nucleotide polymorphism.

Results are ordered by meta-analysis *P*-value. Further details, including minor allele frequencies, are available in Supplementary Data 1.

**Table 2:** Exome array meta-analysis top 10 variants

*CHR*	*Variant*	*rs ID*	*Position*	*A1*	*CLOZUK* P*-value*	*CIAC* P*-value*	*Meta-analysis* P*-value*	*Gene*	*Location*	*Function*
4	exm387558	rs201099591	7 436 363	A	2.48 × 10^−3^	1.31 × 10^−4^	1.10 × 10^−6^	*PSAPL1, SORCS2*	Exonic, intronic	Missense^a^
7	exm622984	rs17139320	63 726 370	G	3.96 × 10^−3^	2.87 × 10^−4^	3.70 × 10^−6^	*ZNF679*	Exonic	Missense^a^
8	exm729894	rs201071539	145 003 862	A	2.19 × 10^−3^	1.69 × 10^−3^	1.19 × 10^−5^	*PLEC*	Exonic	Missense^a^
19	exm1421170	rs2591594	9 076 728	A	4.66 × 10^−3^	3.26 × 10^−3^	4.52 × 10^−5^	*MUC16*	Exonic	Missense^b^
12	exm988839	rs1546308	21 176 135	C	1.25 × 10^−4^	0.0679	9.13 × 10^−5^	*SLCO1B7, SLCO1B3*	Exonic, intronic	Missense^a^
1	exm23767	rs12073549	17 720 545	T	1.60 × 10^−3^	0.0163	9.31 × 10^−5^	*PADI6*	Exonic	Synonymous
15	exm1186658	rs117116488	89 390 513	T	2.76 × 10^−4^	0.0448	9.73 × 10^−5^	*ACAN*	Exonic	Missense^b^
12	exm981950	rs79149293	8 975 873	G	5.66 × 10^−5^	0.0967	1.01 × 10^−4^	*A2ML1*	Exonic	Missense^c^
12	exm995289	rs138912646	42 711 606	A	3.94 × 10^−5^	0.1613	1.79 × 10^−4^	*ZCRB1, PPHLN1*	Exonic, intronic	Missense^a^
12	exm1051374	rs143584336	130 921 539	A	1.22 × 10^−4^	0.1144	2.12 × 10^−4^	*RIMBP2*	Exonic	Missense^a^

Abbreviations: A1, minor reference allele; CHR, chromosome; CIAC, Clozapine-Induced Agranulocytosis Consortium.

Results are ordered by meta-analysis *P*-value. Predicted function of non-synonymous variants.

a Benign.

b Possibly damaging.

c Probably damaging. Further details, including minor allele frequencies, are available in Supplementary Data 3.
