# Erratum: Genome-wide common and rare variant analysis provides novel insights into clozapine-associated neutropenia

**DOI:** 10.1038/mp.2016.137

**Published:** 2016-08-09

**Authors:** S E Legge, M L Hamshere, S Ripke, A F Pardinas, J I Goldstein, E Rees, A L Richards, G Leonenko, L F Jorskog, Clozapine-Induced Agranulocytosis Consortium, K D Chambert, D A Collier, G Genovese, I Giegling, P Holmans, A Jonasdottir, G Kirov, S A McCarroll, J H MacCabe, K Mantripragada, J L Moran, B M Neale, H Stefansson, D Rujescu, M J Daly, P F Sullivan, M J Owen, M C O'Donovan, J T R Walters

**Keywords:** Genome-wide association studies, Genetic variation, Schizophrenia, Adverse effects

**Correction to:** *Molecular Psychiatry* advance online publication, 12 July 2016; doi:10.1038/mp.2016.97

The ninth author’s name was presented incorrectly. It should have been listed as LF Jarskog.

