# Erratum: Genome-wide common and rare variant analysis provides novel insights into clozapine-associated neutropenia

**DOI:** 10.1038/mp.2017.214

**Published:** 2017-10-24

**Authors:** S E Legge, M L Hamshere, S Ripke, A F Pardinas, J I Goldstein, E Rees, A L Richards, G Leonenko, L F Jorskog, Clozapine-Induced Agranulocytosis Consortium, K D Chambert, D A Collier, G Genovese, I Giegling, P Holmans, A Jonasdottir, G Kirov, S A McCarroll, J H MacCabe, K Mantripragada, J L Moran, B M Neale, H Stefansson, D Rujescu, M J Daly, P F Sullivan, M J Owen, M C O'Donovan, J T R Walters

**Keywords:** Genome-wide analysis of gene expression, Molecular neuroscience, Haematological diseases, Drug therapy

**Correction to:** *Molecular Psychiatry* (2017) 22: 1502-1508; advance online publication, 12 July 2017; doi: 10.1038/mp.2016.97

In the first paragraph of the Results section and Figure 1, the authors incorrectly referred to the finding of SNP rs77897117. The correct SNP is rs77897177.

**Figure 1:**
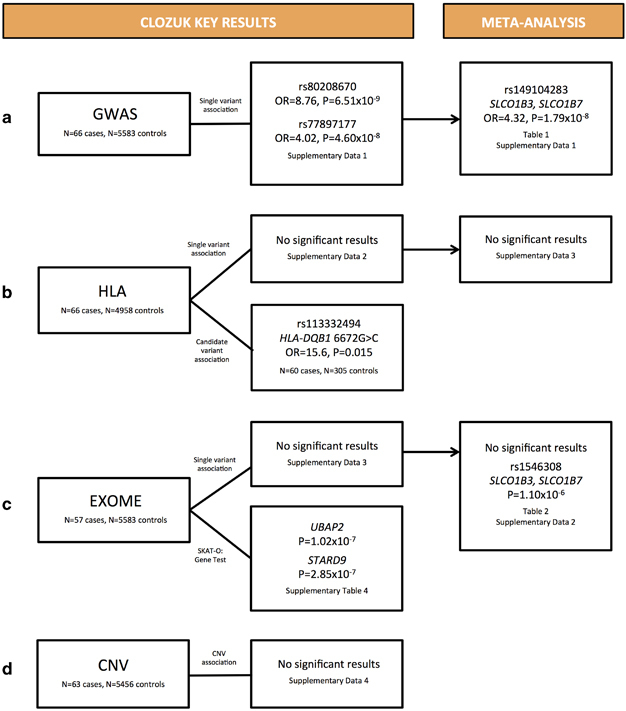
PowerPoint slide

The corrected figure appears in previous page.

## PowerPoint slides

PowerPoint slide for Fig. 1

