# Supplementary material for: Genome-wide common and rare variant analysis provides novel insights into clozapine-associated neutropenia
Source: Mol Psychiatry. 2016 Jul 12;22(10):1502–8. doi: 10.1038/mp.2016.97 (PMC5065090; doi:10.1038/mp.2016.97)
Supplement: Supplementary Information [file mp201697x1.doc]

# Supplementary Information

*Table of Contents*

Supplementary Methods [2](#__RefHeading___Toc309374001)

Sample description [2](#__RefHeading___Toc309374002)

GWAS [2](#__RefHeading___Toc309374003)

Exome array analysis [3](#__RefHeading___Toc309374004)

Copy number variation (CNVs) [4](#__RefHeading___Toc309374005)

Replication gene-based analysis [4](#__RefHeading___Toc309374006)

Supplementary Results [4](#__RefHeading___Toc309374007)

Clozapine-associated neutropenia meta-analyses [5](#__RefHeading___Toc309374008)

Secondary analysis of clozapine-associated neutropenia below ≤ 1000/mm^3^ [5](#__RefHeading___Toc309374009)

Analysis of genotyped variant HLA-DQB1 6672G>C (rs113332494) [6](#__RefHeading___Toc309374010)

Supplementary Tables [9](#__RefHeading___Toc309374011)

Supplementary Table 1: Sample characteristics. [9](#__RefHeading___Toc309374012)

Supplementary Table 2: Proxy SNPs. [9](#__RefHeading___Toc309374013)

Supplementary Table 3: Gene-based SKAT-O analysis from exome chip. [10](#__RefHeading___Toc309374014)

Supplementary Table 4: Predictive test analysis. [10](#__RefHeading___Toc309374015)

Supplementary Figures [11](#__RefHeading___Toc309374016)

Supplementary Figure 1: Principal component analysis of GWAS samples. [11](#__RefHeading___Toc309374017)

Supplementary Figure 2: Principal component analysis of GWAS samples. [12](#__RefHeading___Toc309374018)

Supplementary Figure 3: SNP differences by GWAS genotyping array. [13](#__RefHeading___Toc309374019)

Supplementary Figure 4: Principal component analysis of exome array samples. [14](#__RefHeading___Toc309374020)

Supplementary Figure 5: QQ plot of clozapine-associated neutropenia GWAS. [15](#__RefHeading___Toc309374021)

Supplementary Figure 6: Manhattan plot of clozapine-associated neutropenia GWAS. [16](#__RefHeading___Toc309374022)

Supplementary Figure 7: Clozapine-associated neutropenia GWAS power calculations. [17](#__RefHeading___Toc309374023)

Supplementary Figure 8: QQ plot of clozapine-associated neutropenia exome array analysis. [18](#__RefHeading___Toc309374024)

Supplementary Figure 9: QQ plot of gene-based SKAT-O analysis. [19](#__RefHeading___Toc309374025)

Supplementary Figure 10: QQ plot of clozapine-associated neutropenia ≤ 1000 GWAS. [20](#__RefHeading___Toc309374026)

Supplementary Figure 11: Manhattan plot of clozapine-associated neutropenia ≤ 1000 GWAS. [21](#__RefHeading___Toc309374027)

Supplementary Figure 12: Power calculations for clozapine-associated neutropenia ≤ 1000 GWAS. [22](#__RefHeading___Toc309374028)

Supplementary Figure 13: QQ plot of clozapine-associated neutropenia ≤ 1000 exome array analysis. [23](#__RefHeading___Toc309374029)

Supplementary Figure 14: Association of 12p12.2 with clozapine-associated neutropenia. [24](#__RefHeading___Toc309374030)

Supplementary Figure 15: Principal component analysis of individuals genotyped for rs113332494. [25](#__RefHeading___Toc309374031)

Supplementary Figure 16: ADMIXTURE plot of individuals genotyped for rs113332494. [26](#__RefHeading___Toc309374032)

Supplementary Figure 17: Local ancestry plot of five rs113332494 risk allele carriers. [27](#__RefHeading___Toc309374033)

Supplementary Figure 18: Local ancestry plot of the MHC for CLOZUK sample. [28](#__RefHeading___Toc309374034)

References [29](#__RefHeading___Toc309374035)

# Supplementary Methods

## Sample description

Individuals included in this study were from CLOZUK (N=5493) and CardiffCOGS (Cognition in Schizophrenia, N=156) samples with a clinical or research diagnosis of schizophrenia. ^1^ CLOZUK is comprised of individuals who were prescribed clozapine in the UK and have a clinical diagnosis of treatment-resistant schizophrenia. ^1-3^ The CLOZUK samples were acquired cross-sectionally over a period of three months in collaboration with Novartis Pharmaceuticals, one of the drug companies that provide clozapine (Clozaril®), in accordance with relevant ethics permissions and the UK Human Tissue Act. All individuals were anonymised and only basic demographic details of age, gender, ethnicity and diagnosis were provided. Twelve months after sample acquisition Novartis advised which individuals had developed neutropenia and where available they provided us with the recorded lowest neutrophil counts of these individuals.

CardiffCOGS is a locally recruited schizophrenia sample from South Wales in the UK. ^3,4^ As part of a comprehensive clinical interview, individuals were asked about lifetime clozapine use and occurrence of neutropenia. Clinical case notes were used to confirm neutropenia status and lowest neutrophil levels were collected. Further detailed information of both samples and their ascertainment are described in Rees et al (2014) ^3^.

Clozapine-associated neutropenia cases (N=66, 58 from CLOZUK, 8 from CardiffCOGS) developed an absolute neutrophil count (ANC) ≤ 1500/mm^3^ during treatment with clozapine. Clozapine-treated controls (N=5583, 5435 from CLOZUK, 148 from CardiffCOGS) had received clozapine for a minimum of a year without developing an ANC ≤ 2000/mm^3^. Individuals who had developed an amber result (1500/mm^3^ < ANC ≤ 2000/mm^3^) were excluded from all analyses.

Supplementary Table 1 displays sample characteristics. All individuals included in this study were of European ancestry. A chi-squared test demonstrated no difference in gender between controls and clozapine-associated neutropenia cases (X^2^ = 1.59, P = 0.208). A Mann-Whitney-Wilcoxon test (age not normally distributed) showed no differences in age between controls and clozapine-associated neutropenia cases (P = 0.408).

## GWAS

Genotyping arrays provide an informative backbone of tag single nucleotide polymorphisms (SNPs) thus making large-scale whole-genome genotyping affordable. Quality control procedures and imputation was conducted at the Broad Institute as part of the PGC2 pipeline. ^1^ The quality control parameters for retaining SNPs and subjects were: SNP missingness < 0.05 (before sample removal), subject missingness < 0.02, autosomal heterozygosity deviation (Fhet < 0.2), SNP missingness < 0.02 (after sample removal), and SNP Hardy-Weinberg equilibrium P > 5 x 10^-6^. Imputation was performed using IMPUTE2^5^ (chunk size of 3 Mb and default parameters) with a reference dataset consisting of 2,186 phased haplotypes from the full 1000 Genomes Project dataset (August 2012, 30,069,288 variants, release “v3.macGT1”).

After imputation, SNPs with high imputation quality (INFO > 0.8) and low missingness ( < 0.01) were identified for further quality control. After linkage disequilibrium pruning (r^2^ > 0.02) and frequency filtering (MAF > 0.05), there were 19,551 autosomal SNPs across all 49 datasets of European ancestry in PGC2. This SNP set was used for robust relatedness testing and population structure analysis. Relatedness testing was done with PLINK^6^ and pairs of subjects with 𝜋 > 0.2 were identified and one member of each pair removed at random after preferentially retaining cases over controls. Principal component estimation was conducted to exclude outliers and assess for population stratification using EIGENSTRAT^7^ (Supplementary Figure 1 and 2). To account for subtle population structure or subtle differences in genotyping arrays, the first three principle components and genotyping array were included as covariates in the statistical analyses. SNPs that differed between genotyping arrays at P < 1 x 10^-5^ were excluded (Supplementary Figure 3). We selected common SNPs for analysis with high imputation quality (INFO > 0.8, MAF > 0.01 in cases and controls).

Single variant association with clozapine-associated neutropenia in comparison to clozapine-treated controls, was tested using logistic regression in PLINK^6^ with three principle components and genotyping array included as covariates. PLINK^6^ was used to identify index SNPs in relative linkage equilibrium (--clump-p1 0.0001 --clump-p2 0.0001 --clump-r2 0.1 --clump-kb 3000). The SNP with the most significant association was selected as the index SNP.

## Exome array analysis

The exome array is designed to genotype rare coding variants previously observed in sequencing datasets (http://genome.sph.umich.edu/wiki/Exome_Chip_Design). Quality control procedures were conducted as part of a schizophrenia case control analysis. ^8^ First pass QC was performed using PLINK^6^ on genotypes called using GenCall. Initial QC for probe base exclusions included Hardy-Weinberg equilibrium (HWE) P < 1 x 10^-8^, call rate < 98%, and non-autosomal location. Initial QC for subject exclusions were based on call rate < 98%, as well as relatedness based on identity by descent (IBD) analysis (PI-HAT > 0.1), heterozygosity, and principal component analysis. Principal component estimation was conducted using EIGENSTRAT^7^ based on 3022 SNPs with MAF > 1%. The sample and healthy controls were merged with 1100 samples from 11 populations using the HapMap 3 dataset ^9^, and outliers that did not cluster near to the HapMap European individuals were removed to minimize ancestral heterogeneity. In total we excluded 1204 individuals and 16841 markers prior to the zCall post-processing procedure.

zCall is a post-processing step designed to improve the calling of SNVs. ^10^ We applied zCall to batches using batch-specific intensity data. Markers were subsequently excluded if they were monomorphic, had call rates < 99%, had HWE P < 10^-6^ in any batch, or had a difference in call rate between batches > 1%. We also excluded probes where the allele frequencies differed between the two groups of healthy controls (blood donors versus 1958 Birth cohort) at P < 0.001, or between the schizophrenia cases assayed on the two types of chip at P < 0.0005. These p-value thresholds were derived from QQ-plots of the within healthy control and within schizophrenia case analyses. Lastly, we excluded variants that did not show a sufficient difference in mean intensity between different genotype clusters (GenTrain score < 0.4, cluster separation metric < 0.08).

We applied a further round of QC to the individuals based on the Z-call genotypes, excluding samples on the basis of call rate (>99% required for inclusion), heterozygosity (separately for variants above and below 1% MAF) and concordance between database and genetically determined sex. The final dataset comprised 141,204 variants and data was available for 57 clozapine-associated neutropenia cases and 4958 clozapine-treated controls.

Principal component analysis was conducted using EIGENSTRAT^7^ with 14,743 independent common exome array SNPs (MAF ≥ 0.05 and pruned by r^2^ < 0.2) to assess population structure and identify outliers (Supplementary Figure 4). Due to the relatively small case sample size in our study we did not apply a frequency filter to variants in this analysis.

Logistic regression was conducted in PLINK^6^ to test each variant for association with clozapine-associated neutropenia in comparison to clozapine-treated controls. The first 10 principal components were included as covariates to account for any subtle population structure. Adaptive permutations (between 10 and 1 x 10^9^) were used to provide p-values in the logistic regression analysis due to inflated logistic regression statistics at small allele frequencies in comparison to a Fisher’s exact test or permuted p-values. PLINK was used to identify index SNPs in relative linkage equilibrium (--clump-p1 0.01 --clump-p2 0.01 --clump-r2 0.1 --clump-kb 3000). The SNP with the highest association was selected as the index SNP. Due to reduced reliability of genotype calling for rare variants, all exome array variants noted in this paper were subject to visual inspection of cluster plots.

Variants were allocated to genes according the RefSeq database. In order to test for cumulative effects of rare functional variants in clozapine-associated neutropenia, SKAT-O ^11^ was conducted for genes with at least two rare (MAF < 0.05), functional (non-synonymous, stop or splice) variants. The SKATBinary function within SKAT was utilised with 2x10^6^ permutations and 10 principal components.

## Copy number variation (CNVs)

The identification and quality control of copy number variation (CNV) in the current sample has been previously described in Rees et al 2014 ^3^. CNVs were called using the PennCNV algorithm ^12^ and samples were excluded if they were outliers for the following CNV quality control metrics: log R ratio standard deviation, B-allele frequency drift, wave factor and total number of CNVs. CNVs called in the same individual were joined together if the distance separating them was less than 50% of their combined length. CNVs were excluded if they were called using fewer than 10 probes, were less than 100KB in length, overlapped segmental duplications by more than 50% of their length, had a probe density of < 1 probe per 20Kb or had a frequency > 1%.

Samples that passed both CNV and GWAS QC (63 cases and 5456 controls) were used to test each RefSeq gene for enrichment for exon disrupting CNVs in cases using a 2-sided Fisher’s exact test. Deletions and duplications were analysed separately and P-values were adjusted for multiple testing by applying a Bonferroni correction of 20,000 genes.

## Secondary analysis of clozapine-associated neutropenia below ≤ 1000/mm^3^

We conducted secondary analyses on a subset of the more severely affected cases with ANC ≤ 1000/mm^3^ (n=18, all CLOZUK). A chi-squared test demonstrated no difference in gender between controls and clozapine-associated neutropenia cases below ≤ 1000/mm^3^ (X^2^ = 2.03, P = 0.363). A Mann-Whitney-Wilcoxon test showed no differences in age between controls and clozapine-associated neutropenia below ≤ 1000/mm^3^ (P = 0.622).

We assessed the association of single variants with clozapine-associated neutropenia below ≤ 1000/mm^3^ in GWAS (N=18), exome array (N=16) and HLA imputation (N=18) analyses. All analyses conducted were consistent with methods used for clozapine-associated neutropenia, described above.

## Replication gene-based analysis

Due to differing analytical methods used by CIAC, it was not possible to combine our gene-based results in a joint analysis. CIAC used a Fisher’s exact test to assess the number of carriers of a functional variant whereas we assessed the frequency of variants.

# Supplementary Results

## Clozapine-associated neutropenia meta-analyses

### GWAS

The 1561 SNPs associated with clozapine-associated neutropenia at P < 1 x 10^-4^ were reduced to a subset of 266 independent regions (r^2^ < 0.1), each represented by the most significant SNP. Within the replication sample, data was available for 256 of the 266 top SNPs in our study and a proxy was available for a further SNP. Supplementary Table S2 indicates the proxy SNP used and its LD with the original SNP.

The rs149104283 allele frequencies in the main text are based on imputed dosage (probability) data and it can be useful to consider how this translates to risk allele carrier status. One approach is to consider as risk allele carriers those with a greater probability of being risk allele heterozygotes or homozygotes than their probability of being non-risk allele homozygotes. Applying this to our data we have a total of 9 risk allele carriers in the 66 CLOZUK cases (1.00 median probability of being a carrier) and 14 carriers out of 162 cases in the CIAC sample (0.98 median probability).

### Exome array analysis

The 1290 variants associated with clozapine-associated neutropenia at P < 0.01 were reduced to a subset of 1138 independent regions (r^2^ < 0.1). Data was available for 1023 of the 1138 associated SNPs in the replication sample. However, due to rarity of the variants in this analysis, only 279 variants had a computable odds ratio (OR) in both samples; it was common for the minor allele to be absent from either cases or controls. Although we used a p-value based method for the meta-analysis, the OR is required to determine the direction of effect. Thus, only these 279 variants could be included.

### Imputed HLA analysis

Data was available for 102 of the 139 variants associated with clozapine-associated neutropenia at P < 0.05 in the replication sample.

## Secondary analysis of clozapine-associated neutropenia below ≤ 1000/mm^3^

### GWAS

A total of 6,231,759 genotyped and imputed variants were included in secondary analyses of a subset of the more severely affected cases with ANC ≤ 1000/mm^3^ (n=18) (QQ plot in Supplementary Figure 10, lambda = 0.97). Two intronic loci reached GWS (Manhattan plot, Supplementary Figure 11); rs76415963 in *SLX4IP* on chromosome 20 (OR =18.05, 95% CI: 6.79-48.00, P = 6.63 x 10^-9^) and rs138818969 in *FAM228A* on chromosome 2 (OR = 15.55, 95% CI: 5.99-40.32, P = 1.67 x 10^-8^). Our sample size for clozapine-associated neutropenia ≤ 1000/mm^3^ analyses had 80% power to detect an odds ratio > 10 for alleles with MAF > 0.20 at P < 5 x 10^-8^ (Supplementary Figure 12). Data was available within the replication sample for 361 of the 373 independent SNPs (from 1856 SNPs, r^2^ < 0.1) associated with clozapine-associated neutropenia ≤ 1000/mm^3^ at P < 1 x 10^-4^ and proxies were available for a further three SNPs (Supplementary Table 2). No SNP reached GWS in a meta-analysis. Associations of rs76415963 in *SLX4IP* and rs138818969 in *FAM228A* were not replicated in CIAC (OR = 0.57, P = 0.31 and OR = 0.94, P = 0.97, respectively). The most significantly associated SNP from the meta-analysis was rs143888465 in *SCN8A* on chromosome 12 (OR = 4.94, P = 3.72 x 10^-6^).

### Exome array analysis

An association study of genotyped exome array variants (N=114,814) in a subset of 16 cases with neutropenia ≤ 1000/mm^3^ did not identify any loci exceeding GWS threshold of P < 4.3 x 10^-7^, corresponding to a Bonferroni correction for 115,000 variants tested (QQ plot in Supplementary Figure 13, lambda = 0.94). The 1296 variants associated with clozapine-associated neutropenia ≤ 1000/mm^3^at P < 0.01 were reduced to a subset of 1110 independent regions (r^2^ < 0.1). Data was available for 990 of these variants in CIAC. However, due to rarity of the variants in this analysis, only 291 variants had an odds ratio (OR) in both samples; it was common for the minor allele to be absent from either cases or controls. Because the OR is required to determine the direction of effect in meta-analyses, only these 291 variants could be included in our meta-analysis. No variant exceeded a significance threshold of P < 4.3 x 10^-7^. The most significant variant was rs17139320 (P = 7.56 x 10^-6^), a missense variant in *ZNF679* and present in 12.5% of cases vs. 2.96% of controls in the CLOZUK sample and 7.41% of cases vs. 2.67% of controls in the CIAC sample.

### Imputed HLA analysis

An additional analysis of a clozapine-associated neutropenia ≤ 1000/mm^3^ in 6,672 imputed variants did not identify any loci exceeding GWS. The most significant variant was rs3129963 (OR = 4.14, P = 3.94 x 10^-5^), located 5kb upstream of *BTNL2* and present in 44.44% of cases and 16.94% of controls. *BTNL2* was implicated in CIAC analysis ^13^. Data was available within CIAC for 763 of the 839 *HLA* variants associated with clozapine-associated neutropenia ≤ 1000/mm^3^ at P < 0.05. No imputed classical *HLA* allele or amino acid polymorphism was associated at GWS in the combined meta-analysis. rs3129963, 5kb from *BTNL2*, was weakly associated in CIAC (OR = 1.32, P = 0.048). The most significant variant from the meta-analysis was rs3129891 (OR = 1.91, P = 6.28 x 10^-8^), located 2kb downstream of *HLA-DRA* and present in 44.44% of cases and 18.83% of controls in the CLOZUK sample and 29.93% of cases and 19.97% of controls in the CIAC sample. Among the top associated variants in the meta-analysis were amino acid changes in *HLA-B*, a gene been previously implicated in clozapine-induced agranulocytosis ^13^. Due to complex linkage disequilibrium (LD) in this region, independent SNPs were not identified. Thus it is likely that the variants listed do not represent independent signals.

## Analysis of genotyped variant HLA-DQB1 6672G>C (rs113332494)

Association with clozapine-associated neutropenia was tested using Fisher’s exact test given low minor allele counts but to ensure there were no effect of population stratification, we also conducted a logistic regression including three PCAs derived from GWAS with 5 x 10^8^ permutations to generate empirical p-values. After adjusting for GWAS PCAs, rs113332494 remained associated with clozapine-associated neutropenia (OR = 12.4, 95% CI: 1.12 - 137.4, P = 0.015) and neutropenia ≤ 1000/mm^3^ (OR = 22.5, 95% CI: 1.01 - 502.4, P = 0.039).

Given the candidate design, the analyses of rs13332494 were limited to samples of European ancestry. However, if further population-based exclusions were applied, one of the heterozygote carriers would be classed as an outlier (Supplementary Figure 15). When this individual is excluded from the analyses, the association with clozapine-associated neutropenia attenuates (OR = 10.5, 95% CI: 0.94-116.7, P = 0.0699) and neutropenia ≤ 1000/mm^3^ (OR = 19.7, 95% CI: 1.2-321.5, P = 0.0973). We conducted admixture analyses to address the appropriateness of excluding this sample and also present wider population-based analyses relevant to this genetic variant and region. We found no evidence to suggest that past admixture had caused a strong differentiation of the MHC region in this individual in comparison to other individuals with the same HLA genotype or to the wider population to which this individual belongs.

### Population stratification

It has been recently proposed that recombination profiles in the MHC region differ greatly between human continental populations ^14^, which supports the increased genetic diversity shown by admixed individuals in HLA loci ^15,16^. Usually, in research initiatives undertaken in European populations, admixed individuals and other outliers are removed as part of standard quality-control procedures if whole-genome information is available ^17^. However, careful evaluation of the ancestry patterns of well-genotyped outliers, especially when their similarity to the main population cluster is high, can contribute to the identification and interpretation of novel genetic association signals ^18,19^. Therefore, we assessed the population structure in our panel of 347 individuals for which the rs113332494 SNP (HLA-DQB1 region) was genotyped, by combining their whole-genome genotypes with the publicly available 1000 Genomes Project samples ^20^. In this combined dataset, the EIGENSTRAT software was used with recommended settings ^21^, including the removal of long-range LD regions, which includes the MHC ^22^. A plot of the first two principal components showed the CLOZUK samples as closely related to the EUR superpopulation cluster, particularly to the GBR and CEU populations (Supplementary Figure 15).

### Ancestry profiling

One of the CLOZUK samples was shown to be a population outlier. In the full PCA plot, this individual is close to some admixed populations, such as Colombians and Mexicans (not shown). To obtain direct estimates of ancestry for this individual, we ran the ADMIXTURE software ^23,24^ in the 347 CLOZUK individuals with default parameters and K=3 (Supplementary Figure 16). The resulting three ancestral components were allocated to a specific ancestry group by comparing their distributions in the CEU, CHB and YRI 1000 Genomes populations, although the algorithm was run in unsupervised mode. This analysis showed a complex ancestry profile for the population outlier individual, in which past admixture from East Asian and West African populations had left a minor, but noticeable, signature (~5% of the genome for each).

### Local ancestry analysis of the HLA-DQB1 locus

To detect the influence of subtle admixture in the HLA-DQB1 locus, we applied the local ancestry inference algorithm ELAI ^25^ to the four individuals that were heterozygote for the rs113332494 SNP. For increased accuracy tailored to our purposes, we ran the analysis on the entire extended MHC region xMHC: 26 to 34 Mb of chromosome 6^; 26^, using the 1000 Genomes super populations EUR, EAS and AFR as proxies for Western European, East Asian and West African ancestry respectively. For defining the main algorithm parameters, we used the recommended settings for three ancestral populations (S=3, K=15), and a mean ancestral tract length of γ=200. In the genomic map of chromosome 6, this corresponds roughly to 0.5 cM, which is common for the Identity-by-Descent (IBD) segments found in the xMHC region ^27^. The algorithm inferred almost complete East Asian ancestry in the HLA-DQB1 region on the population outlier individual (Supplementary Figure 17), but also detected EAS/AFR admixture in another two heterozygotes, one of them a control without neutropenia.

### Population-wide local ancestry analysis of the xMHC region

In order to assess the prevalence of these admixture patterns in our schizophrenia sample, we applied the ELAI algorithm with the same parameters on the entire CLOZUK sample. The resulting average profile (Supplementary Figure 18) shows that the prevalence of Western European ancestry diminishes along the xMHC region, a feature previously shown in other populations ^28,29^ that is thought to be a consequence of the positive selective pressures experienced by HLA alleles. In particular, we noted an increase of East Asian ancestry in the Class III and Class II HLA clusters the latter including DQB1^; 30^. However, this finding is seen at consistent rate in the cases and controls (Supplementary Figure 18).

We also note that in the 1000 genomes data the frequency of the associated allele at rs113332494 is 0% (monomorphic) in the 1KG British (GBR) population sample and 1.01% in the European (CEU) population sample.

# Supplementary Tables

|  | **Male gender**  **N (%)** | **Mean age (range)** |
| --- | --- | --- |
| Controls (N=5617) | 4016 (71.5) | 41.9 (16-90) |
| Clozapine-associated neutropenia cases (N=66) | 40 (60.6) | 40.3 (20-64) |

## Supplementary Table 1: Sample characteristics.

Gender and age of clozapine-associated neutropenia cases and clozapine-treated controls.

| **SNP** | **CHR** | **BP** | **Proxy SNP** | **Distance (bp)** | **r^2^** | **D’** |
| --- | --- | --- | --- | --- | --- | --- |
| *Clozapine-associated neutropenia* | | | | | | |
| rs116216021 | 6 | 3417882 | rs116552069 | 11430 | 0.96 | 1 |
| *Clozapine-associated neutropenia ≤ 1000* | | | | | | |
| rs111698467 | 8 | 25624468 | rs113539283 | 84249 | 0.807 | 0.992 |
| rs111242461 | 10 | 37404727 | chr10_37367098_I | 37629 | 0.840 | 0.932 |
| rs76415963 | 20 | 10544516 | rs74762384 | 7575 | 0.814 | 1 |

## Supplementary Table 2: Proxy SNPs.

Proxy SNPs used for clozapine-associated neutropenia and clozapine-associated neutropenia ≤ 1000 meta-analyses. Columns are: SNP = name of original variant identified in CLOZUK analysis; CHR = chromosome; BP = base pair position (hg19); Proxy SNP = proxy SNP identified within CIAC; Distance (bp) = distance between original and proxy SNP; r^2^ = LD between original and proxy SNP; D’ = LD between original and proxy SNP.

| **Gene** | **N variants** | **MAC controls**  **(n=5415)** | **MAC cases (n=57)** | **Method** | **P-value** |
| --- | --- | --- | --- | --- | --- |
| *UBAP2* | 4 | 37 | 7 | QA | 1.02 x 10^-7^ |
| *STARD9* | 10 | 776 | 30 | UA | 2.85 x 10^-7^ |
| *ITFG3* | 3 | 10 | 4 | ER | 6.57 x 10^-6^ |
| *CLHC1* | 3 | 597 | 11 | UA | 9.53 x 10^-6^ |
| *GRIK3* | 3 | 5 | 3 | ER | 1.92 x 10^-5^ |
| *AKAP9* | 8 | 167 | 10 | QA | 2.27 x 10^-5^ |
| *PAK6* | 5 | 187 | 10 | QA | 4.38 x 10^-5^ |
| *SMARCAD1* | 4 | 169 | 8 | QA | 8.76 x 10^-5^ |
| *LYST* | 8 | 263 | 13 | QA | 1.39 x 10^-4^ |
| *ASTN2* | 5 | 382 | 12 | QA | 1.46 x 10^-4^ |

## Supplementary Table 3: Gene-based SKAT-O analysis from exome chip.

Top 10 associated genes with clozapine-associated neutropenia from SKAT-O analysis of cumulative effects of rare functional variants within genes. Columns are: GENE = gene name; N variants = number of variants contributing to gene; MAC controls = total count of rare functional alleles within gene in controls; MAC controls = total count of rare functional alleles within gene in cases; Method = Analysis method (Efficient resampling (ER); Quantile adjusted moment matching (QA); No adjustment (UA)); P-value = p-value for gene.

|  | **rs149104283** | **rs1546308** | **rs149104283 & rs1536308** | **rs113332494** | **rs149104283, rs1536308 & rs113332494** |
| --- | --- | --- | --- | --- | --- |
| N Cases | 64 | 56 | 55 | 60 | 48 |
| N Controls | 5391 | 4924 | 4832 | 305 | 245 |
| Sensitivity | 10.94 | 28.57 | 30.91 | 5.00 | 29.17 |
| Specificity | 97.61 | 88.57 | 89.26 | 99.67 | 90.61 |
| PPV | 13.97 | 8.15 | 9.28 | 35.13 | 9.94 |
| NPV | 96.86 | 97.22 | 97.32 | 96.73 | 97.30 |

## Supplementary Table 4: Predictive test analysis.

Analysis of utility of identified variants as a predictive test, based on a prevalence of 3.43% (for agranulocytosis and neutropenia combined). Rows are: N Cases = number of cases in analysis; N Controls = number of controls in analysis; Sensitivity; Specificity; PPV = positive predictive value; NPV = negative predictive value. Columns refer to variants included in analysis: rs149104283 = GWS intronic variant; rs1546308 = missense variant; rs113332494 = HLA-DQB1 6672G>C.

# Supplementary Figures


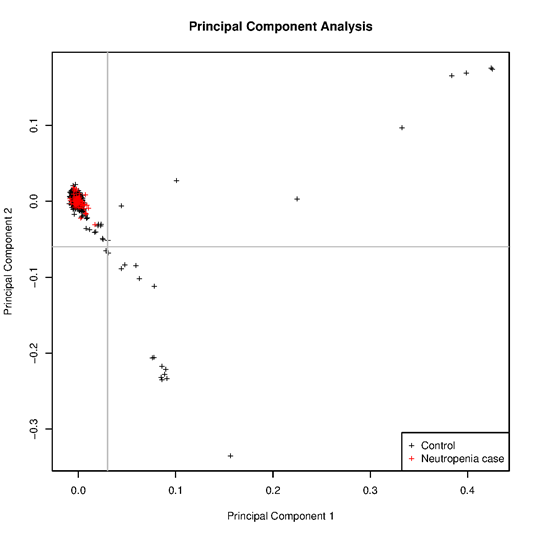


## Supplementary Figure 1: Principal component analysis of GWAS samples.

Principal component analysis conducted using EIGENSTRAT to assess and control for population structure in GWAS. Figure displays principal component 1 and 2. Points represent individual samples; black points represent clozapine-treated controls and red point represent neutropenia cases. Grey lines represent exclusions (top left corner included).


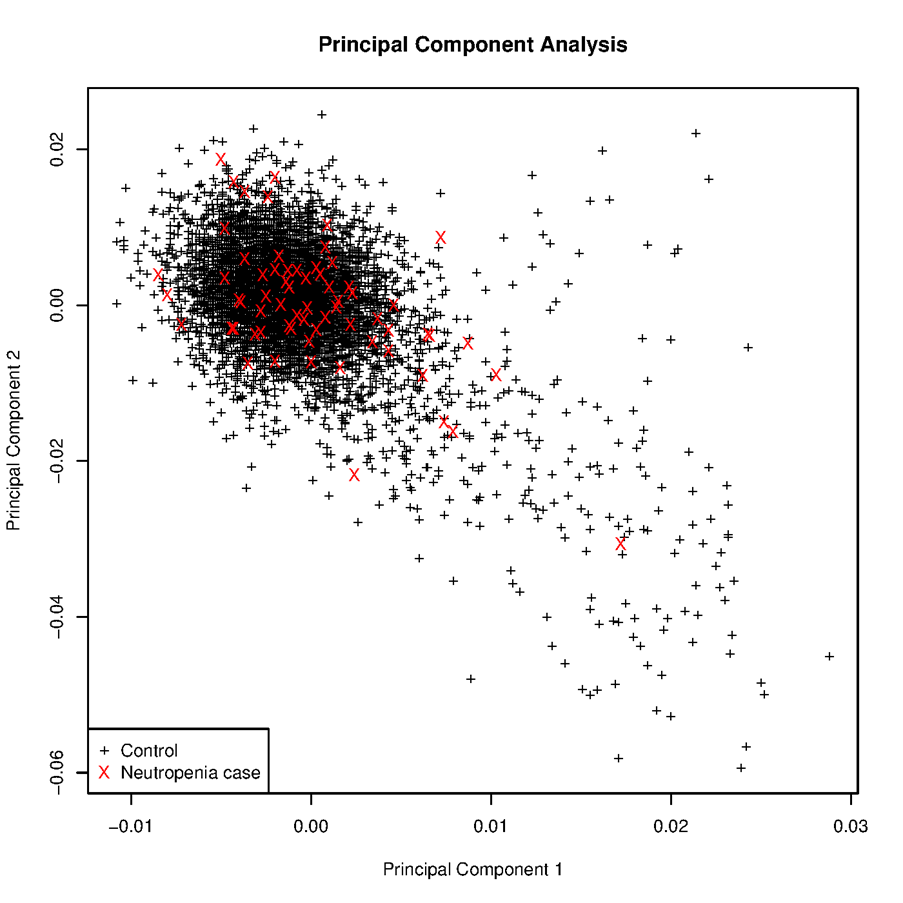


## Supplementary Figure 2: Principal component analysis of GWAS samples.

Close-up of Supplementary Figure 1. Figure displays principal component 1 and 2. Points represent individual samples included in GWAS analysis; black points represent clozapine-treated controls and red point represent neutropenia cases.


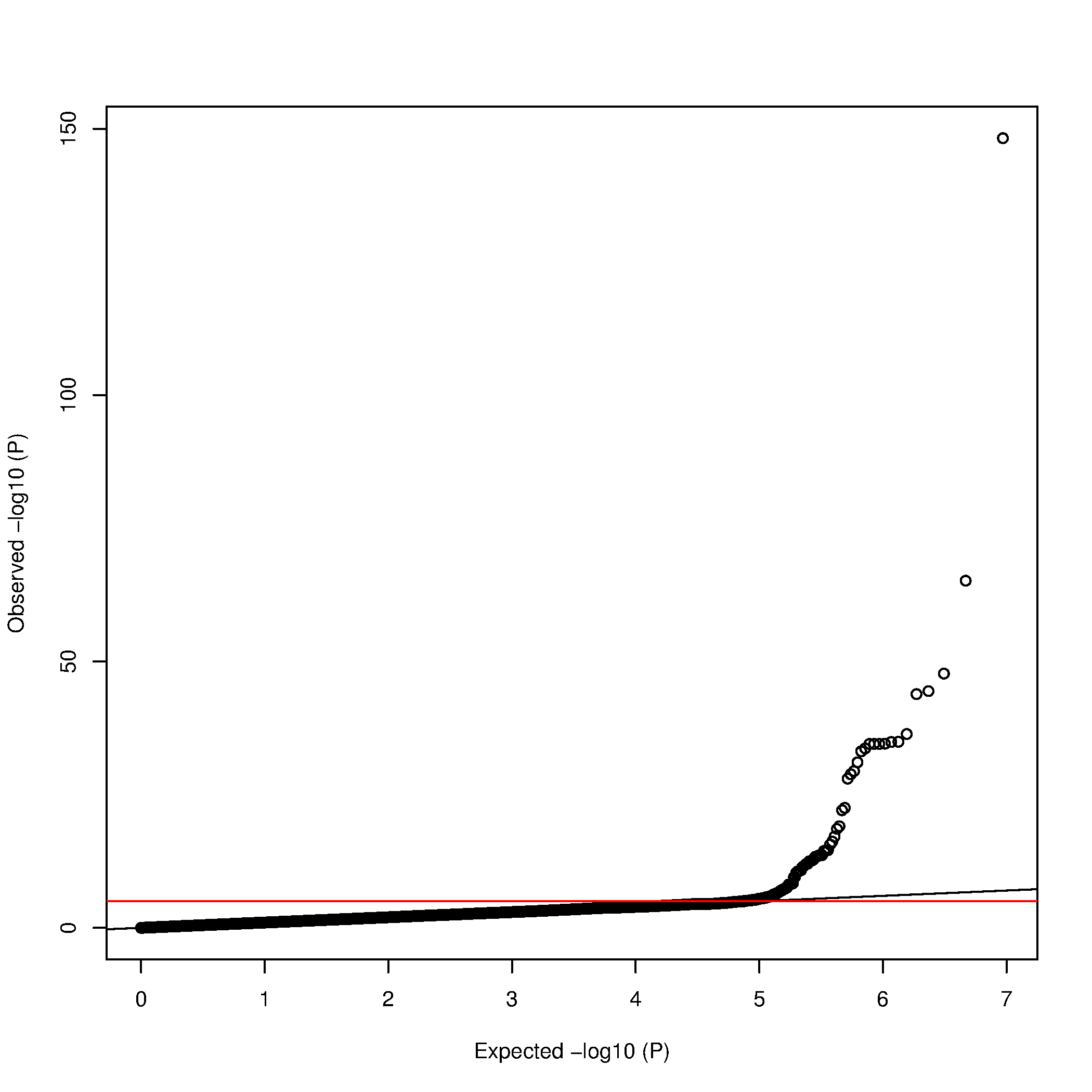


## Supplementary Figure 3: SNP differences by GWAS genotyping array.

QQ plot of GWAS comparing SNPs genotyped on HumanOmniExpressExome-8v1 and Illumina HumanOmniExpress-12v1. Logistic regression was conducted in PLINK to detect SNPS that performed differently between genotyping arrays. 124 SNPs with P < 1 x 10^-5^, represented by the red line, were excluded from GWAS analyses. Genotyping array was included as a covariate in order to account for subtle SNP differences.


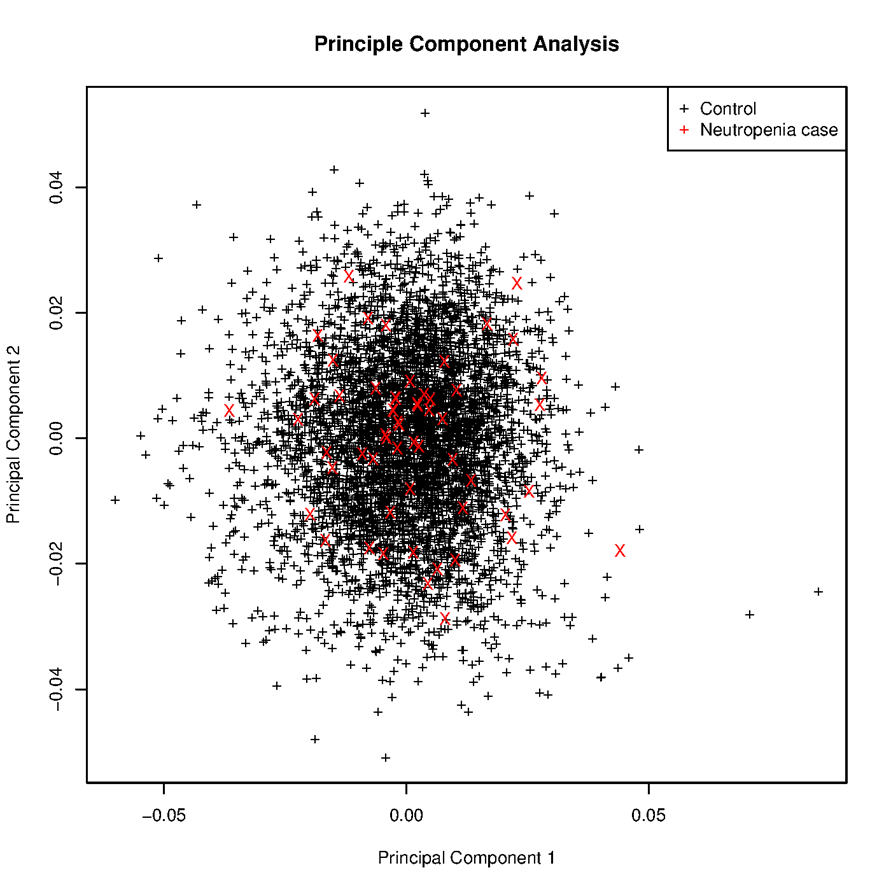


## Supplementary Figure 4: Principal component analysis of exome array samples.

Principal component analysis conducted using EIGENSTRAT to assess and control for population structure in exome array analysis. Figure displays principal component 1 and 2 for each individual included in exome array analysis; black points represent clozapine-treated controls and red points represent neutropenia cases.


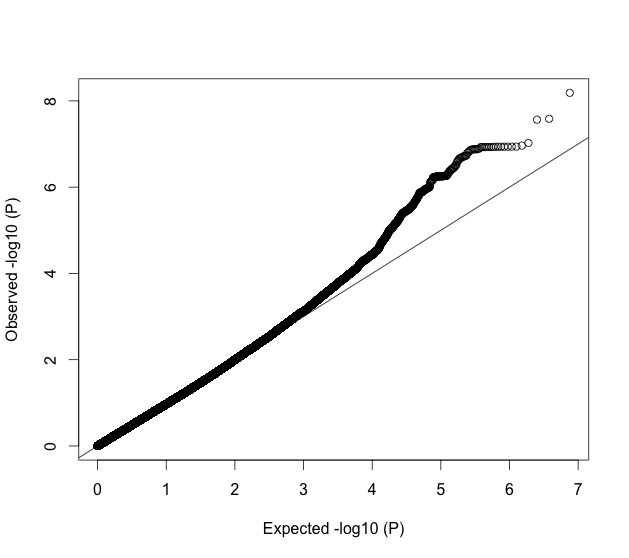


## Supplementary Figure 5: QQ plot of clozapine-associated neutropenia GWAS.

QQ plot of clozapine-associated neutropenia GWAS of genotyped and imputed variants. The –log10 observed logistic regression p-values (y-axis) are plotted against expected p-values (x-axis). λ_GC_ = 0.95.

##
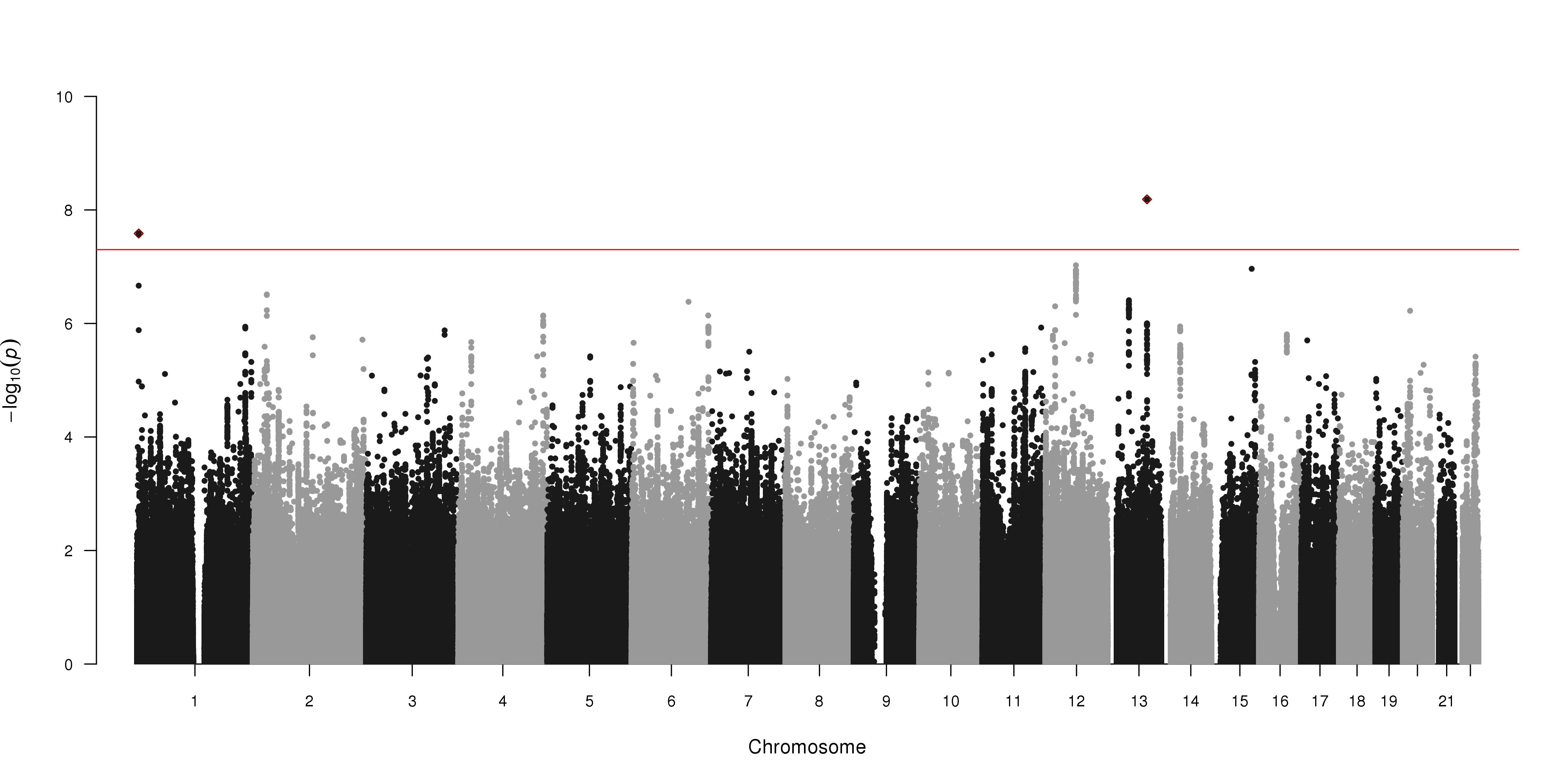
Supplementary Figure 6: Manhattan plot of clozapine-associated neutropenia GWAS.

–log10 P-values (y-axis) for each SNP is presented on the basis of chromosomal position (x axis). The red line represents genome wide significance level (P < 5 x 10^-8^).

## Supplementary Figure 7: Clozapine-associated neutropenia GWAS power calculations.

Power calculations derived from Quonto software (http://biostats.usc.edu/Quanto.html) with additive disease model at a disease prevalence of 3% for 66 Cases and 5619 Controls (1:85 ratio) to detect genome wide significant variant (p = 5 x 10^-8^) at minor allele frequencies of 0.1 (red), 0.2 (orange), 0.3 (pink), 0.4 (blue) and 0.5 (green) for effect sizes (OR) 1-10. The sample size in clozapine-associated neutropenia genome-wide common variants analysis was 80% powered to detect an OR > 4 for MAF > 0.10.

## Supplementary Figure 8: QQ plot of clozapine-associated neutropenia exome array analysis.

The –log10 observed permuted logistic regression p-values (y-axis) are plotted against expected p-values (x-axis). λ_GC_ = 1.11.


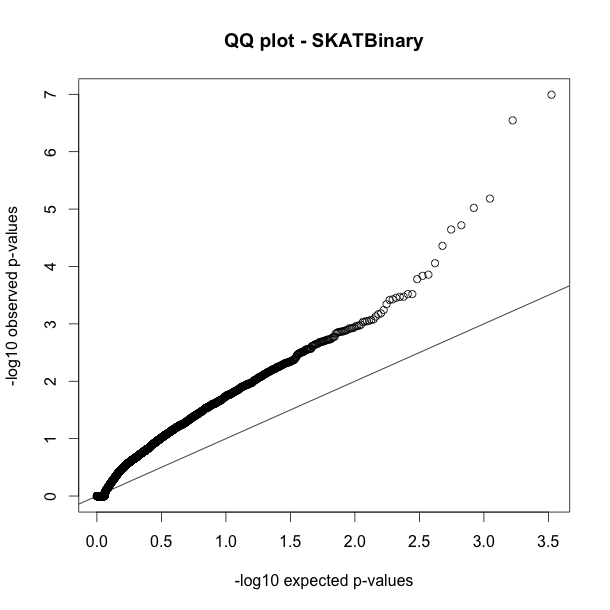


## Supplementary Figure 9: QQ plot of gene-based SKAT-O analysis.

The –log10 observed permuted SKAT-O p-values (y-axis) for each gene is plotted against expected p-values (x-axis). λ_GC_ = 3.39.


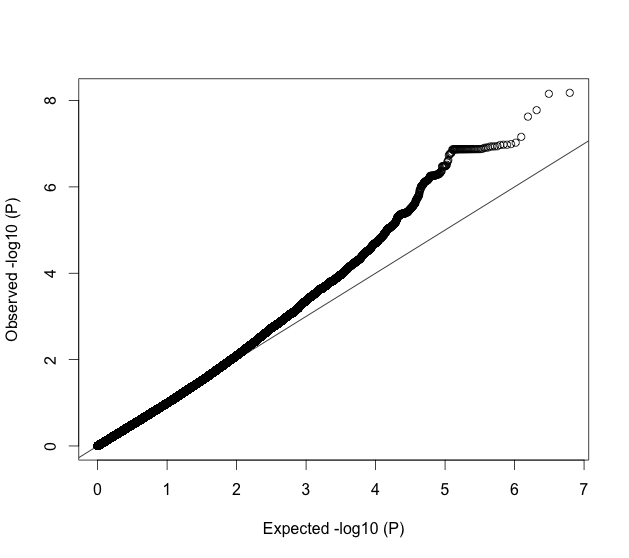


## Supplementary Figure 10: QQ plot of clozapine-associated neutropenia ≤ 1000 GWAS.

The –log10 observed logistic regression p-values (y-axis) are plotted against expected p-values (x-axis). λ_GC_ = 0.97.

##
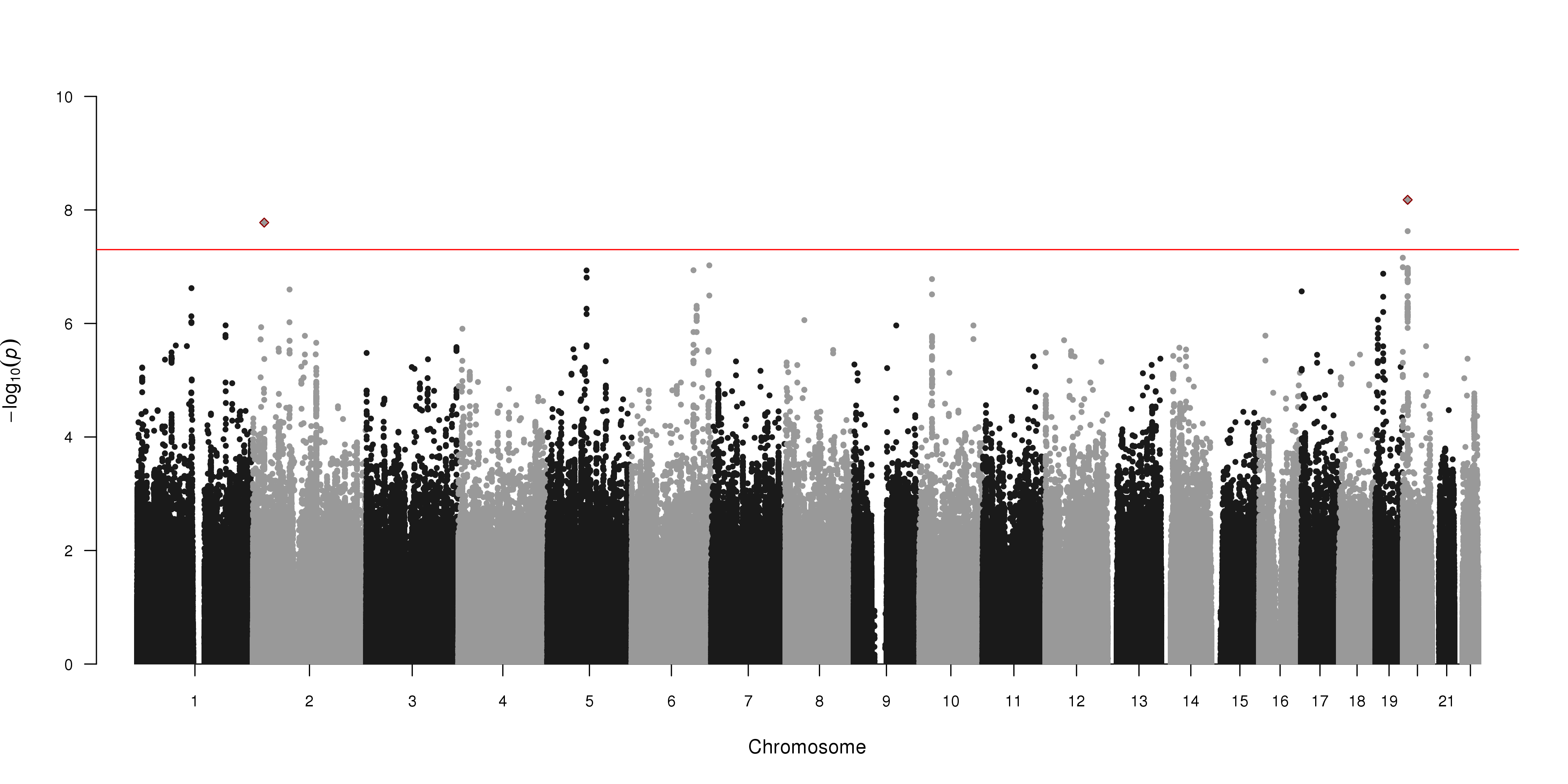
Supplementary Figure 11: Manhattan plot of clozapine-associated neutropenia ≤ 1000 GWAS.

–log10 P-values (y-axis) for each SNP is presented on the basis of chromosomal position (x axis). The red line represents genome wide significance level (P < 5 x 10^-8^).

## Supplementary Figure 12: Power calculations for clozapine-associated neutropenia ≤ 1000 GWAS.

Power calculations derived from Quonto software (http://biostats.usc.edu/Quanto.html) with additive disease model at a disease prevalence of 1% for 18 Cases and 5619 Controls (1:312 ratio) to detect genome wide significant variant (p = 5x10^-8^) at minor allele frequencies of 0.1 (red), 0.2 (orange), 0.3 (pink), 0.4 (blue) and 0.5 (green) for effect sizes (OR) 1-20. The sample size in clozapine-associated neutropenia ≤ 1000 genome-wide common variants analysis was 80% powered to detect an OR > 10 for MAF > 0.20.

## Supplementary Figure 13: QQ plot of clozapine-associated neutropenia ≤ 1000 exome array analysis.

QQ plot of clozapine-associated neutropenia below ≤ 1000 exome array analysis. The –log10 observed permuted logistic regression p-values (y-axis) are plotted against expected p-values (x-axis). λ_GC_ = 0.94.

## Supplementary Figure 14: Association of 12p12.2 with clozapine-associated neutropenia.

LocusZoom plot of the region associated with clozapine-associated neutropenia on chromosome 12p12.2 from meta-analysis. Genes within the region are shown in the lower panel, and the unbroken blue line indicated the recombination rate within the region. Each circle represents the P-value for one SNP in the meta-analysis, with the top SNP rs149104283 shown in purple and the SNPs in the region coloured depending on their degree of correlation (r^2^) with rs149104283 (as estimated by LocusZoom on the basis of CEU HapMap haplotypes).


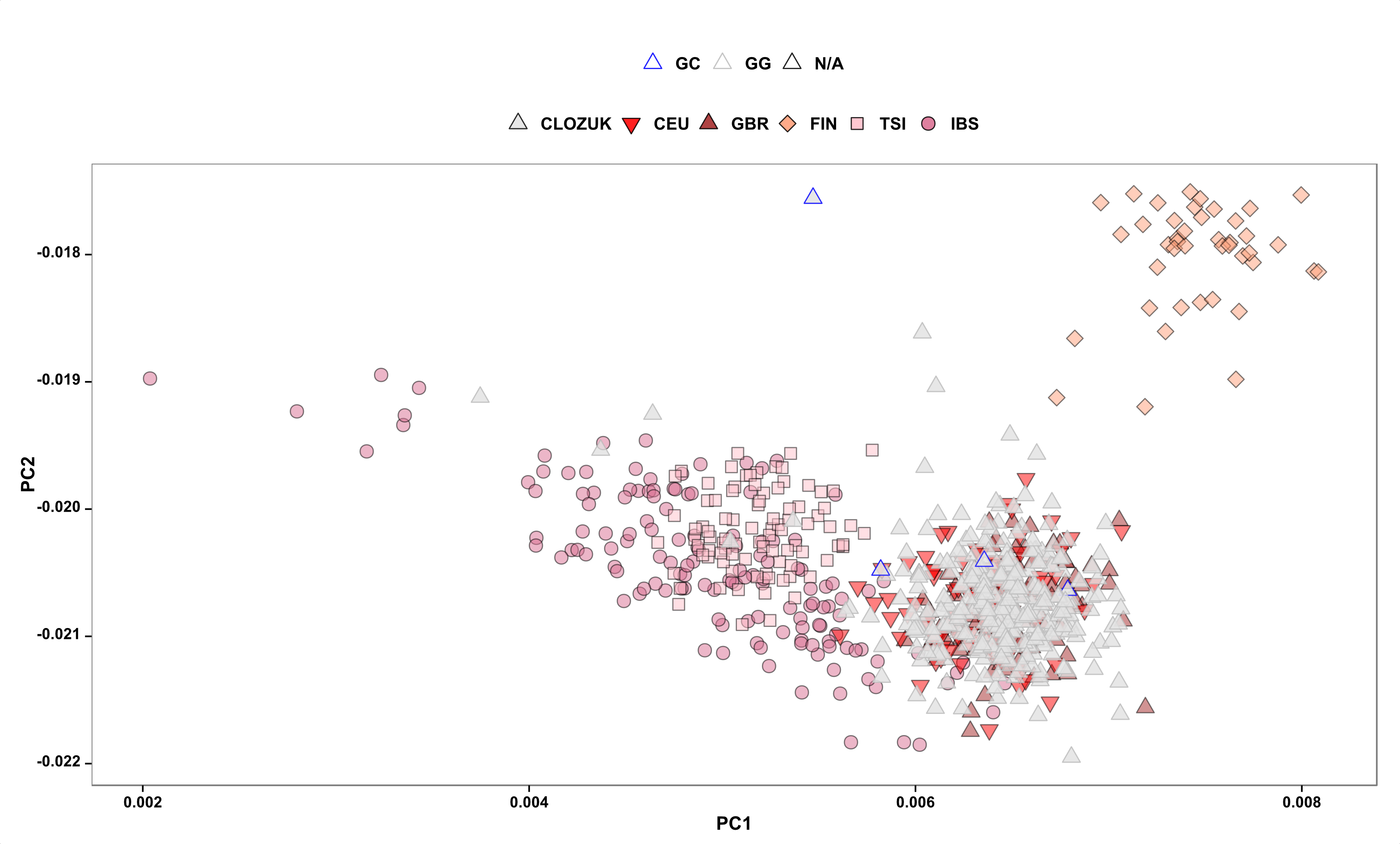


## Supplementary Figure 15: Principal component analysis of individuals genotyped for rs113332494.

Principal component analysis of the 347 CLOZUK individuals genotyped for rs113332494 and the 1000 Genomes Populations. An individual colour shows its population while the colour of its border shows the genotyping status. This detail views shows only populations pertaining to the EUR group, as all of the CLOZUK individuals fall between their boundaries. Notice that one of the heterozygote individuals (10N668761; arrow) is an outlier in respect of the other CLOZUK samples.


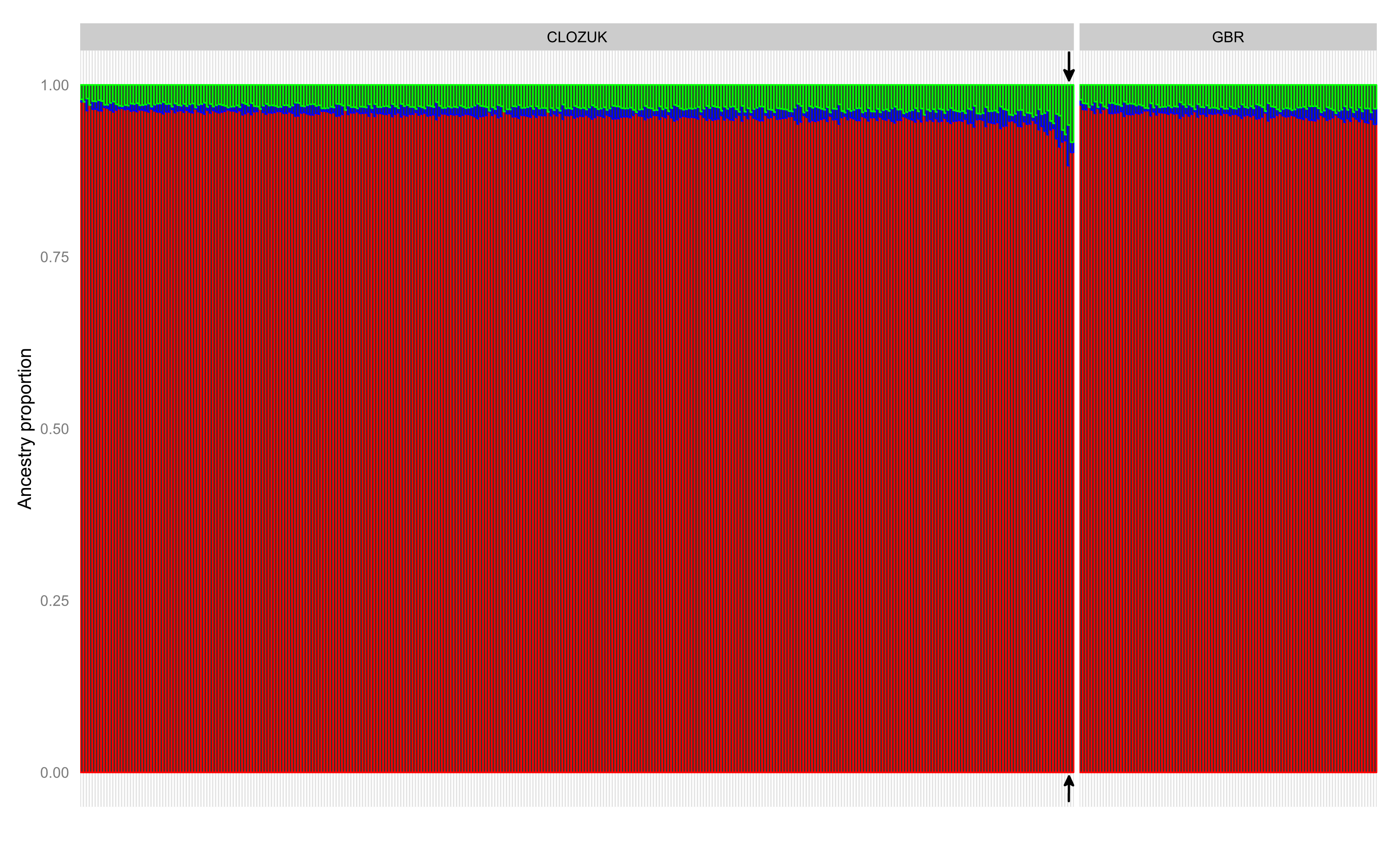


## Supplementary Figure 16: ADMIXTURE plot of individuals genotyped for rs113332494.

ADMIXTURE plot of the 347 CLOZUK individuals genotyped for rs113332494 inferring K=3 ancestral components. Each bar shows the admixture profile of a single individual, with Western European ancestry shown in red, East Asian ancestry shown in blue and West African ancestry shown in green. The admixture profile of the 10N668761 individual (second to last; arrow) is 88.26% Western European, 5.91% East Asian and 5.82% West African. A profiling of the GBR 1000 Genomes Population using the same parameters is shown at the right side of the plot for comparison.


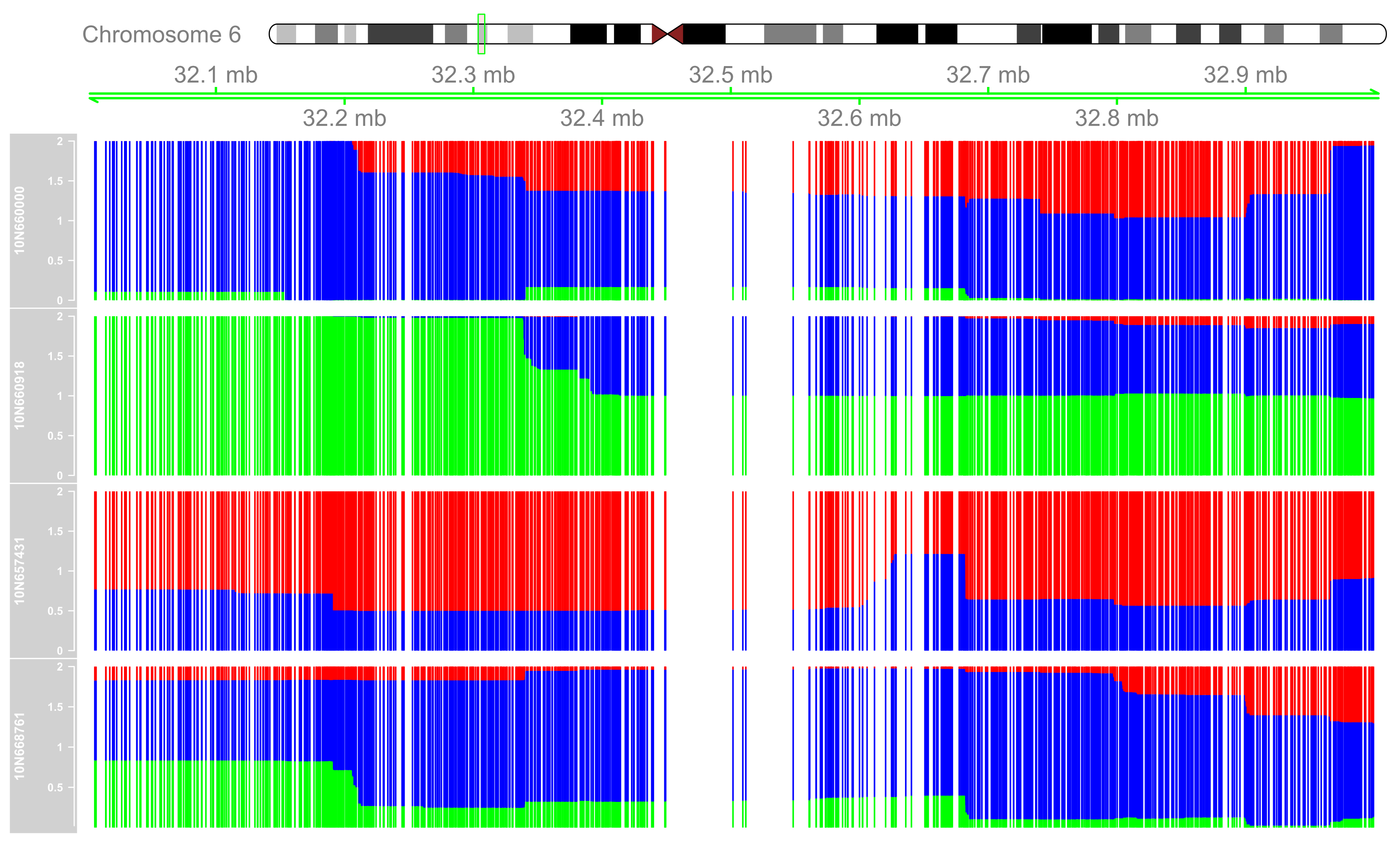


## Supplementary Figure 17: Local ancestry plot of five rs113332494 risk allele carriers.

Local ancestry plot of four individuals that are G/C heterozygote for rs113332494 in HLA-DQB1 (32.63-32.64 Mb) and surrounding xMHC regions. With the exception of 10N660000, all have clozapine-associated neutropenia. Scale bars indicate the number (0, 1, 2) of alleles inferred as contributed by an ancestral population, with EUR shown in red, EAS shown in blue and AFR shown in green. All estimations are averages over 10 replicates of the ELAI algorithm. White bars indicate areas in which ancestry inference was not possible due to the OmniExpress chip having no coverage.


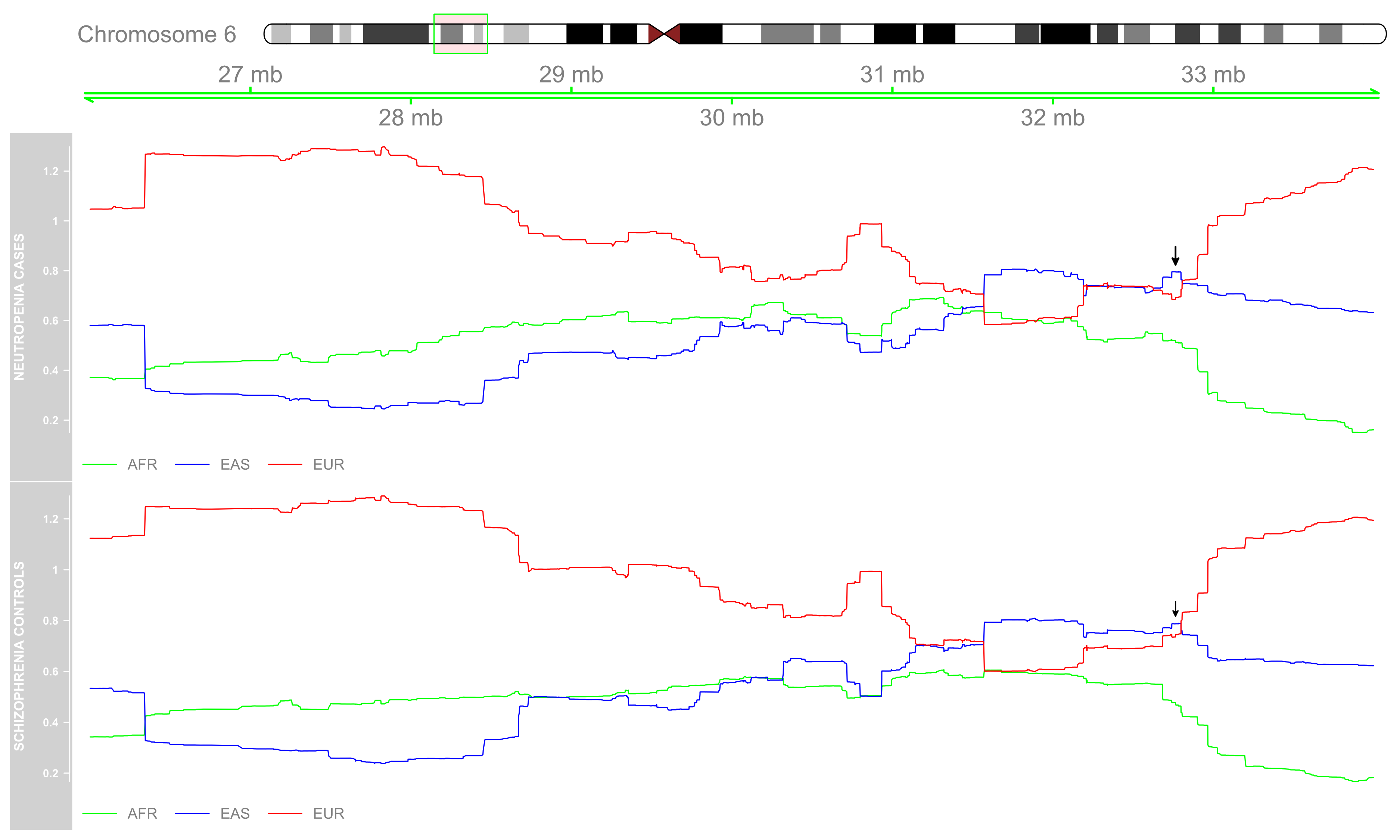


## Supplementary Figure 18: Local ancestry plot of the MHC for CLOZUK sample.

Local ancestry plot of the extended MHC region for the entire CLOZUK population. Lines indicate the average number (0, 1, 2) of alleles inferred as contributed by an ancestral population, and areas without direct genotype coverage were interpolated. Notice the increase of ASN ancestry in the HLA-DQB1 locus (arrows) for both groups relative to surrounding loci. For each individual used in the plot, all estimations are averages over 10 replicates of the ELAI algorithm. The black arrow indicates the position of the associated HLADQB1 SNP rs113332494. Controls are those with schizophrenia on clozapine who have not developed neutropenia.

# References

1. Schizophrenia Working Group of the Psychiatric Genomics Consortium. Biological insights from 108 schizophrenia-associated genetic loci. *Nature* 2014; **511**: 421-427.

2. Hamshere, M.L., Walters, J.T., Smith, R., Richards, A.L., Green, E., Grozeva, D. *et al.* Genome-wide significant associations in schizophrenia to ITIH3/4, CACNA1C and SDCCAG8, and extensive replication of associations reported by the Schizophrenia PGC. 2012.

3. Rees, E., Walters, J.T., Georgieva, L., Isles, A.R., Chambert, K.D., Richards, A.L. *et al.* Analysis of copy number variations at 15 schizophrenia-associated loci. *Br J Psychiatry* 2014; **204**: 108-114.

4. Carroll, L.S., Williams, H.J., Walters, J., Kirov, G., O'Donovan, M.C. & Owen, M.J. Mutation screening of the 3q29 microdeletion syndrome candidate genes DLG1 and PAK2 in schizophrenia. *Am J Med Genet B Neuropsychiatr Genet* 2011; **156B**: 844-849.

5. Howie, B.N., Donnelly, P. & Marchini, J. A flexible and accurate genotype imputation method for the next generation of genome-wide association studies. *PLoS Genet* 2009; **5**: e1000529.

6. Purcell, S., Neale, B., Todd-Brown, K., Thomas, L., Ferreira, M.A., Bender, D. *et al.* PLINK: a tool set for whole-genome association and population-based linkage analyses. *Am J Hum Genet* 2007; **81**: 559-575.

7. Price, A.L., Patterson, N.J., Plenge, R.M., Weinblatt, M.E., Shadick, N.A. & Reich, D. Principal components analysis corrects for stratification in genome-wide association studies. *Nat Genet* 2006; **38**: 904-909.

8. Richards, A.L., Leonenko, G., Walters, J.T., Kavanagh, D.H., Rees, E.G., Evans, A. *et al.* Exome arrays capture polygenic rare variant contributions to schizophrenia. *Human molecular genetics* 2016.

9. Ripke, S., O'Dushlaine, C., Chambert, K., Moran, J.L., Kahler, A.K., Akterin, S. *et al.* Genome-wide association analysis identifies 13 new risk loci for schizophrenia. *Nat Genet* 2013; **45**: 1150-1159.

10. Goldstein, J.I., Crenshaw, A., Carey, J., Grant, G.B., Maguire, J., Fromer, M. *et al.* zCall: a rare variant caller for array-based genotyping: genetics and population analysis. *Bioinformatics* 2012; **28**: 2543-2545.

11. Lee, S., Emond, M.J., Bamshad, M.J., Barnes, K.C., Rieder, M.J., Nickerson, D.A. *et al.* Optimal unified approach for rare-variant association testing with application to small-sample case-control whole-exome sequencing studies. *Am J Hum Genet* 2012; **91**: 224-237.

12. Wang, K., Li, M., Hadley, D., Liu, R., Glessner, J., Grant, S.F. *et al.* PennCNV: an integrated hidden Markov model designed for high-resolution copy number variation detection in whole-genome SNP genotyping data. *Genome Res* 2007; **17**: 1665-1674.

13. Goldstein, J.I., Fredrik Jarskog, L., Hilliard, C., Alfirevic, A., Duncan, L., Fourches, D. *et al.* Clozapine-induced agranulocytosis is associated with rare HLA-DQB1 and HLA-B alleles. *Nature Commun* 2014; **5**: 4757.

14. Lam, T.H., Shen, M., Chia, J.M., Chan, S.H. & Ren, E.C. Population-specific recombination sites within the human MHC region. *Heredity* 2013; **111**: 131-138.

15. Levin, A.M., Adrianto, I., Datta, I., Iannuzzi, M.C., Trudeau, S., McKeigue, P. *et al.* Performance of HLA allele prediction methods in African Americans for class II genes HLA-DRB1,-DQB1, and-DPB1. 2014; **15**: 72.

16. Zúñiga, J., Yu, N., Barquera, R., Alosco, S., Ohashi, M., Lebedeva, T. *et al.* HLA Class I and Class II Conserved Extended Haplotypes and Their Fragments or Blocks in Mexicans: Implications for the Study of Genetic Diversity in Admixed Populations. 2013; **8**: e74442.

17. Weale, M. Quality Control for Genome-Wide Association Studies. in *Genetic Variation*, Vol. 628 (eds. Barnes, M.R. & Breen, G.) 341-372 (Humana Press, 2010).

18. Culverhouse, R.C., Hinrichs, A.L. & Suarez, B.K. Identifying cryptic population structure in multigenerational pedigrees in a Mexican American sample. in *BMC proceedings* Vol. 8 S4 (BioMed Central Ltd, 2014).

19. Torgerson, D.G., Gignoux, C.R., Galanter, J.M., Drake, K.A., Roth, L.A., Eng, C. *et al.* Case-control admixture mapping in Latino populations enriches for known asthma-associated genes. 2012; **130**: 76-82.e12.

20. 1000 Genomes Project Consortium. An integrated map of genetic variation from 1,092 human genomes. 2012; **491**: 56-65.

21. Price, A.L., Patterson, N.J., Plenge, R.M., Weinblatt, M.E., Shadick, N.A. & Reich, D. Principal components analysis corrects for stratification in genome-wide association studies. 2006; **38**: 904-909.

22. Price, A.L., Weale, M.E., Patterson, N., Myers, S.R., Need, A.C., Shianna, K.V. *et al.* Long-range LD can confound genome scans in admixed populations. 2008; **83**: 132.

23. Alexander, D.H., Novembre, J. & Lange, K. Fast model-based estimation of ancestry in unrelated individuals. 2009; **19**: 1655-1664.

24. Alexander, D.H. & Lange, K. Enhancements to the ADMIXTURE algorithm for individual ancestry estimation. 2011; **12**: 246.

25. Guan, Y. Detecting structure of haplotypes and local ancestry. 2014; **196**: 625-642.

26. de Bakker, P.I.W., McVean, G., Sabeti, P.C., Miretti, M.M., Green, T., Marchini, J. *et al.* A high-resolution HLA and SNP haplotype map for disease association studies in the extended human MHC. 2006; **38**: 1166-1172.

27. Albrechtsen, A., Moltke, I. & Nielsen, R. Natural selection and the distribution of identity-by-descent in the human genome. 2010; **186**: 295-308.

28. Shriner, D., Adeyemo, A., Ramos, E., Chen, G. & Rotimi, C.N. Mapping of disease-associated variants in admixed populations. 2011; **12**: 223.

29. Ordoñez, G., Romero, S., Orozco, L., Pineda, B., Jiménez-Morales, S., Nieto, A. *et al.* Genomewide admixture study in Mexican Mestizos with multiple sclerosis. 2015; **130**: 55-60.

30. Horton, R., Wilming, L., Rand, V., Lovering, R.C., Bruford, E.A., Khodiyar, V.K. *et al.* Gene map of the extended human MHC. 2004; **5**: 889-899.
