# Supplementary figures and images for: Erratum: Genome-wide common and rare variant analysis provides novel insights into clozapine-associated neutropenia
Source: Mol Psychiatry. 2017 Oct 24;23(1):162–3. doi: 10.1038/mp.2017.214 (PMC5754465; doi:10.1038/mp.2017.214)

## Slide 1
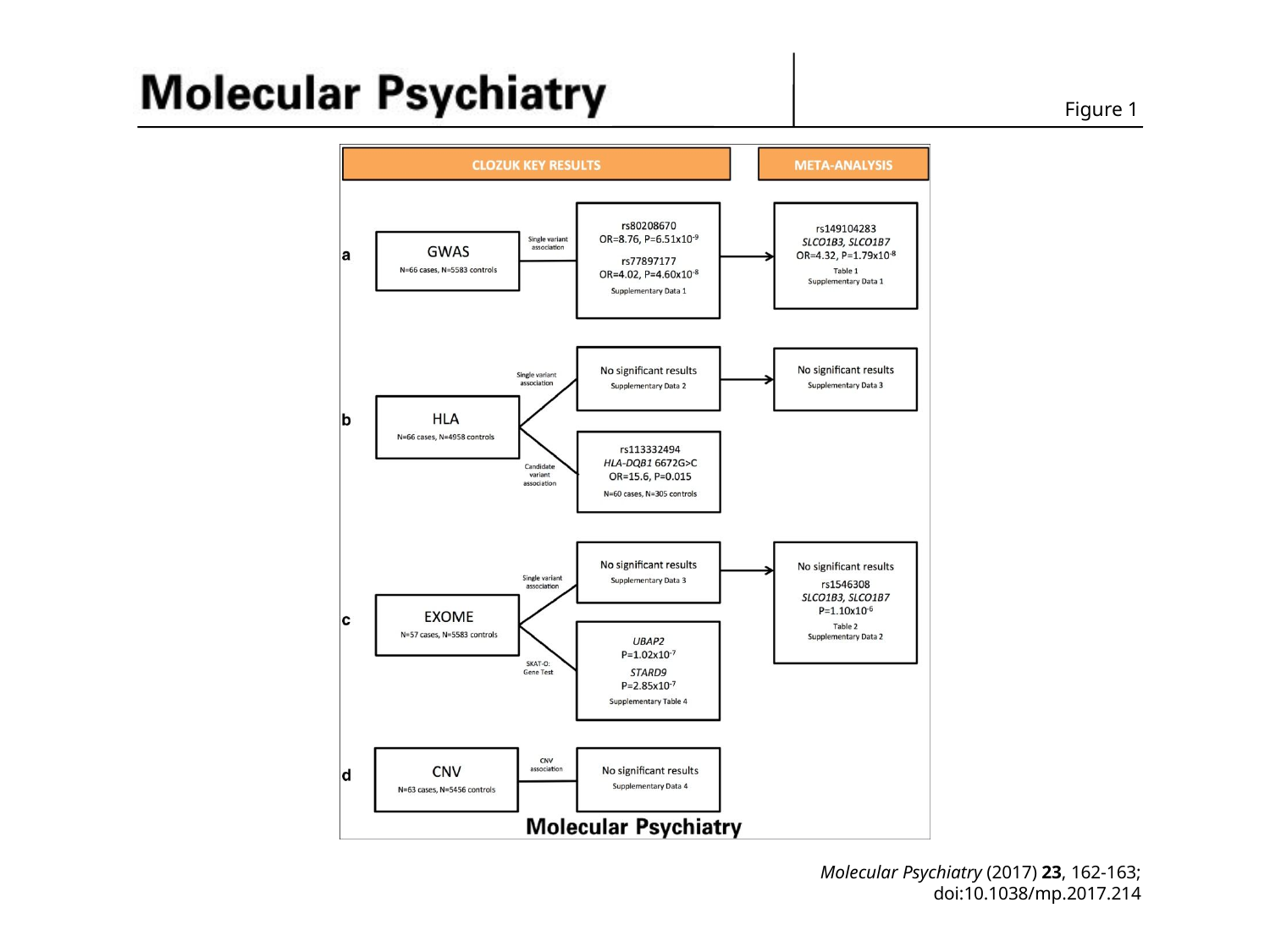

Figure 1
Molecular Psychiatry (2017) 23, 162-163;
doi:10.1038/mp.2017.214

Supplement: Supplementary file 1 — PowerPoint slide for Fig. 1 [file 41380_2018_BFmp2017214_MOESM37_ESM.ppt]
